# Galectin-3 is a non-classic RNA binding protein that stabilizes the mucin *MUC4* mRNA in the cytoplasm of cancer cells

**DOI:** 10.1038/srep43927

**Published:** 2017-03-06

**Authors:** Lucie Coppin, Audrey Vincent, Frédéric Frénois, Belinda Duchêne, Fatima Lahdaoui, Laurence Stechly, Florence Renaud, Céline Villenet, Isabelle Van Seuningen, Emmanuelle Leteurtre, Johann Dion, Cyrille Grandjean, Françoise Poirier, Martin Figeac, Delphine Delacour, Nicole Porchet, Pascal Pigny

**Affiliations:** 1 Univ. Lille, UMR-S 1172 - JPARC - Jean-Pierre Aubert Research Center, F-59000 Lille, France; 2 Inserm, UMR-S 1172, Team “Mucins, Epithelial Differentiation and Carcinogenesis”, F-59000 Lille, France; 3 CHU Lille, Service de Biochimie « Hormonologie, Métabolisme-Nutrition, Oncologie », F-59000 Lille, France; 4 Univ. Lille, RADEME (Research team on rare developmental and metabolic diseases), F-59000 Lille, France; 5 CHU Lille, Service d’anatomie-cytologie-pathologie, F-59000 Lille, France; 6 Univ. Lille, CHU Lille, Plate-forme de génomique fonctionnelle, Centre de Biologie et Pathologie, F-59037 Lille cedex, France; 7 Equipe Ingénierie Moléculaire et Glycobiologie, UMR CNRS 6286, Unité Fonctionnalité et Ingénierie des Protéines, Faculté des Sciences et Techniques, F-44322 Nantes cedex 3, France; 8 Equipe « Morphogenèse, homéostasie et pathologies » Institut Jacques Monod - UMR 7592 CNRS - Université Paris Diderot, F-75205 Paris Cedex 13, France; 9 Equipe CAM « Cell adhesion and mechanics” Institut Jacques Monod - UMR 7592 CNRS - Université Paris Diderot, F-75205 Paris Cedex 13, France

## Abstract

Pancreatic cancer cells express high levels of *MUC1, MUC4 and MUC16* mRNAs that encode membrane-bound mucins. These mRNAs share unusual features such as a long half-life. However, it remains unknown how mucin mRNA stability is regulated. Galectin-3 (Gal-3) is an endogenous lectin playing important biological functions in epithelial cells. Gal-3 is encoded by *LGALS3* which is up-regulated in pancreatic cancer. Despite the absence of a RNA-recognition motif, Gal-3 interacts indirectly with pre-mRNAs in the nucleus and promotes constitutive splicing. However a broader role of Gal-3 in mRNA fate is unexplored. We report herein that Gal-3 increases *MUC4* mRNA stability through an intermediate, hnRNP-L which binds to a conserved CA repeat element in the 3′UTR in a Gal-3 dependent manner and also controls *Muc4* mRNA levels in epithelial tissues of *Gal3*^−/−^ mice. Gal-3 interacts with hnRNP-L in the cytoplasm, especially during cell mitosis, but only partly associates with protein markers of P-Bodies or Stress Granules. By RNA-IP plus RNA-seq analysis and imaging, we demonstrate that Gal-3 binds to mature spliced *MUC4* mRNA in the perinuclear region, probably in hnRNP-L-containing RNA granules. Our findings highlight a new role for Gal-3 as a non-classic RNA-binding protein that regulates *MUC4* mRNA post-transcriptionally.

Galectin-3 (Gal-3), which is a soluble β-galactoside-binding lectin encoded by *LGALS3*, shuttles from the nucleus to the cytosol, and is also secreted in the extracellular milieu by a non-classical secretory pathway^1^,^2^. *In vitro* studies based on cell-free systems, depletion and reconstitution experiments, have demonstrated that Gal-3 is incorporated into the spliceosome complex through its association with the U1 snRNP (small nuclear RiboNucleoProtein) and promotes pre-mRNA splicing^3^,^4^,^5^. Moreover, Gal-3 also interacts with other protein members of the splicing machinery such as Gem associated protein 4 (Gemin-4)^6^. Interactions between Gal-3 and the spliceosome are thought to be mediated by the C-terminal carbohydrate recognition domain (CRD) but also by the N-terminal domain (ND) of Gal-3, especially the YPG-rich repeats^7^. However, the association of Gal-3 with the U1 snRNP is weak and can be disrupted by moderately high K^+^ concentrations^4^. Thus, although Gal-3 is associated with mRNA maturation it can not be considered as a classical RNA-binding protein (RBP) because of the absence of a RNA Recognition Motif (RRM). Moreover, classical RBPs generally influence the fate of mRNA at multiple points during its metabolism, including splicing, nuclear export, storage, stability and/or translation^8^. Apart from the pre-mRNA splicing function of Gal-3, there are no reports to date describing its role in other steps of mRNA metabolism despite its ability to shuttle from the nucleus to the cytosol.

In mammals, Gal-3 exerts a wide range of biological functions. In epithelial cells, it is an important mediator of carcinogenesis, inflammation and fibrosis^9^,^10^. Mice lacking Galectin-3 (*gal*-*3*^−/−^ mice) dysplay defects in intestinal epithelial morphogenesis, with the appearance of microvilli-like structures at the basolateral membrane of enterocytes and defects in the trafficking of components of the microvilli^11^. Furthermore, allergen-challenged *gal*-*3*^−/−^ mice exhibit reduced airway hyper-responsiveness and peri-bronchial fibrosis as well as decreased production of mucus compared with wild-type mice^12^. These abnormalities may be related to the positive, but molecularly uncharacterized, effects of Gal-3 on type 1 collagen, α-smooth muscle actin and fibronectin, as well as on the production of interleukin (IL)-5, IL-12 and TGFβ. However, the molecular mechanisms underlying the effects of Gal-3 on the production of the pulmonary mucins MUC5AC, MUC5B and/or MUC4^13^, which are the main protein components of mucus, have not yet been studied.

Epithelial mucins are high molecular weight glycoproteins with numerous clustered O-glycan chains linked to tandem repeat domains of the peptide backbone. They are classified as secreted (such as MUC2, MUC5AC or MUC5B) or membrane-associated mucins (such as MUC1, MUC4 or MUC16). Epithelial mucins play key roles in epithelial homeostasis (protection, lubrification, pathogen barrier)^14^,^15^. Besides the long extracellular highly glycosylated domain that contributes to the formation of the glycocalyx at the apex of epithelial cells, membrane-associated mucins also have an intracellular short tail enabling contacts with the signaling machinery and/or the actin cytoskeleton. However, in cancer cells, membrane-associated mucins play specific roles. MUC1 and MUC4 activate genes involved in cell proliferation whereas MUC16 promotes cell motility and metastatis^16^,^17^. At the mRNA level, most mucin genes display features that are uncommon among human messengers such as a large size (varying, for example, from 14 to 27 kb for *MUC4* full-length transcript due to allelic variations in the number of tandem repeats)^18^, the presence of a large internal exon and a long half-life (up to 21 h for *MUC4* mRNA in normal bronchial cells^19^). Apart from studies focusing on miRNAs, very few studies have addressed the mechanisms responsible for the “hyper” stability of *MUC* transcripts.

In this study, we searched for novel functions of Gal-3 in the control of mRNA fate using a cellular model depleted in Gal-3 and *gal*-*3*^−/−^ mice. We demonstrated that Gal-3 acts as a post-trancriptional regulator of *MUC4* mRNA through interacting with and enhancing hnRNP-L binding and activation of a CA repeat element (CARE) present in human, mouse and rat *Muc4* 3′UTR. We also showed that Gal-3 is able to bind to mature spliced *MUC4* mRNAs at the perinuclear region, in RNA granules distinct from P-Bodies or Stress Granules.

## Results

### *MUC4* mRNA is stabilized by Galectin-3

**Figure 1:**
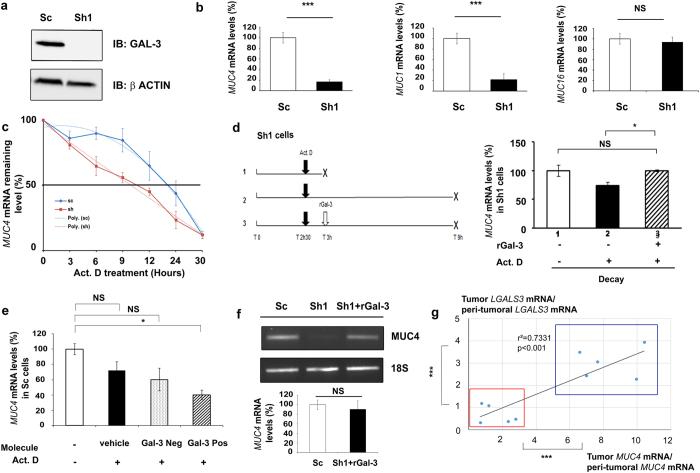
Galectin-3 increases *MUC4* mRNA stability by a post-transcriptional mechanism. (**a**) Gal-3 expression was analysed in whole cell protein extracts by western blotting. β-actin was used as a loading control. Blots are representative of three independent experiments. (**b**) *MUC4, MUC1* and *MUC16* mRNA levels were quantified in Sc and Sh1 cells by RT-qPCR. *18S* RNA was used as an internal control. Results (mean ± SD) are expressed in comparison with the mRNA levels present in Sc cells (arbitrarily set at 100%); n = 3. (**c**) Decay curves of *MUC4* mRNA in Sc and Sh1 cells cultured in the presence of 8 μg/ml actinomycin-D (Act. D) for the indicated times. Values represent percentage of the remaining mRNA levels. Interpolating spline curve appeared in dotted line; n = 3. (**d**) Right, Decay of *MUC4* mRNA levels assessed by RT-qPCR on a 6 h period time of an Act. D treatment (between T 3 h and T 9 h) in presence or absence of rGal-3. Crosses on figure indicate mRNA collection. Results (mean ± SD) are expressed in comparison with *MUC4* mRNA level present in condition 1 (arbitrarily set at 100%); n = 3. Left, scheme describing the experimental procedure. (**e**) Sc cells were treated for 6 h with 200 μM of a Galectin-3 competitive inhibitor (Gal-3 Pos), a structurally relative negative control (Gal-3 Neg) or vehicle in association with Act. D. *MUC4* mRNA and *18S* RNA were quantified by absolute qPCR. Results (mean ± SD) are expressed in comparison with *MUC4* mRNA level present in condition without Act. D (arbitrarily set at 100%); n = 2. (**f**) Influence of a rGal-3 treatment in Sh1 cells (36 h, 4 μM) on *MUC4* mRNA levels assessed by RT-PCR analysis (*18S* RNA as an internal control, n = 3) Densitometric analysis of the bands was performed using ImageQuanTL software. (**g**) Total RNAs were extracted from 10 human pancreatic ductal adenocarcinomas (cancer (tumoral) and surrounding healthy (peri-tumoral) pancreatic tissues). *MUC4, LGALS3* and 18S RNA were quantified by RT-qPCR. Results are expressed as an induction factor of *MUC4* and *LGALS3* expression in comparison with the corresponding healthy pancreatic tissue (mRNA level arbitrarily set at 1). *p < 0.05; ***p < 0.001; NS: not significant by Student’s *t*-test.

Sh1 cells are derived from CAPAN-1 pancreatic cancer cell line where Gal-3 was knockdown using a shRNA approach^20^. Gal-3 silencing was confirmed by western blotting using Sc cells as controls (Fig. 1a). RT-qPCR analysis showed that Sh1 cells expressed lower levels of *MUC1* and *MUC4* mRNAs than the control Sc cells whereas *MUC16* mRNA levels did not vary (Fig. 1b), suggesting that Gal-3 positively controls the expression of *MUC1* and *MUC4* either at a transcriptional or post-transcriptional level. Transient co-transfections of Sh1 cells with different constructs generated to express a luciferase reporter gene under the control of the *MUC1 or MUC4* promoters did not reveal any positive and significant effects of Gal-3 at the transcriptional level (Fig. S1). To determine the potential of Gal-3 to regulate the mRNA half-life, we blocked transcription with actinomycin D (Act. D) and measured the mRNA levels by RT-qPCR in Sc and Sh1 cells. The half-life of *MUC4* transcripts was 22.3 h (±1.6 h) in Sc cells, whereas it decreased to 11.3 h (±0.5 h) in Sh1 cells (*p* < 0.01; Fig. 1c). By contrast, *MUC1* mRNA half-life, which was around 9.8 h (±2.7 h), was not significantly influenced by Gal-3 (not shown). Finally, *MUC16* mRNA was particularly stable (half-life >30 h); therefore its decay rate could not be determined accurately in this study (not shown). Next, we evaluated the effects of recombinant Gal-3 (rGal-3) treatment on Act. D treated Sh1 cells (Fig. 1d). 6 h Act. D treatment duration was chosen since it was the first time point associated with a significant reduction of *MUC4* mRNA levels in Sh1 cells versus Sc cells (p < 0.05) based on the decay curve (Fig. 1c). Addition of rGal-3, which is known to be internalized^21^, slowed the decay of *MUC4* mRNA in Sh1 cells (Fig. 1d, *p* < 0.05). Conversely, treatment of Sc cells with a specific competitive inhibitor of Gal-3 CRD in the presence of Act. D for 6 h decreased *MUC4* mRNA levels (Fig. 1e). Moreover, internalization of rGal-3 in Sh1 cells restored *MUC4* mRNA levels comparable to controls (Fig. 1f). Altogether these results show that Gal-3 specifically stabilizes *MUC4* transcripts by a post-transcriptional mechanism. To evaluate the relevance of our cellular model findings in human cancer, we quantified *MUC4* and *LGALS3* mRNA levels in the cancerous and non-tumoral areas obtained from 10 human pancreatic ductal adenocarcinomas (PDAC). A significant correlation was identified between *MUC4* mRNA levels and *LGALS3* mRNA levels (*p* < 0.001, Fig. 1g). Interestingly, the samples could be separated into two subgroups that differed significantly in terms of expression levels: a first one characterized by low *LGALS3* & low *MUC4* and a second one with high *LGALS3* & high *MUC4* mRNA levels. Such a correlation was not found (Fig. S2) among the same samples between *LGALS3* and *MUC1* or *MUC16* mRNAs, which are commonly overexpressed in PDAC^20^,^22^, thus confirming the specificity of the *MUC4*-*LGALS3* relationship in this type of epithelial cancer.

### Galectin-3 prevents *MUC4* mRNA decay in cooperation with hnRNP-L in epithelial tissues

**Figure 2:**
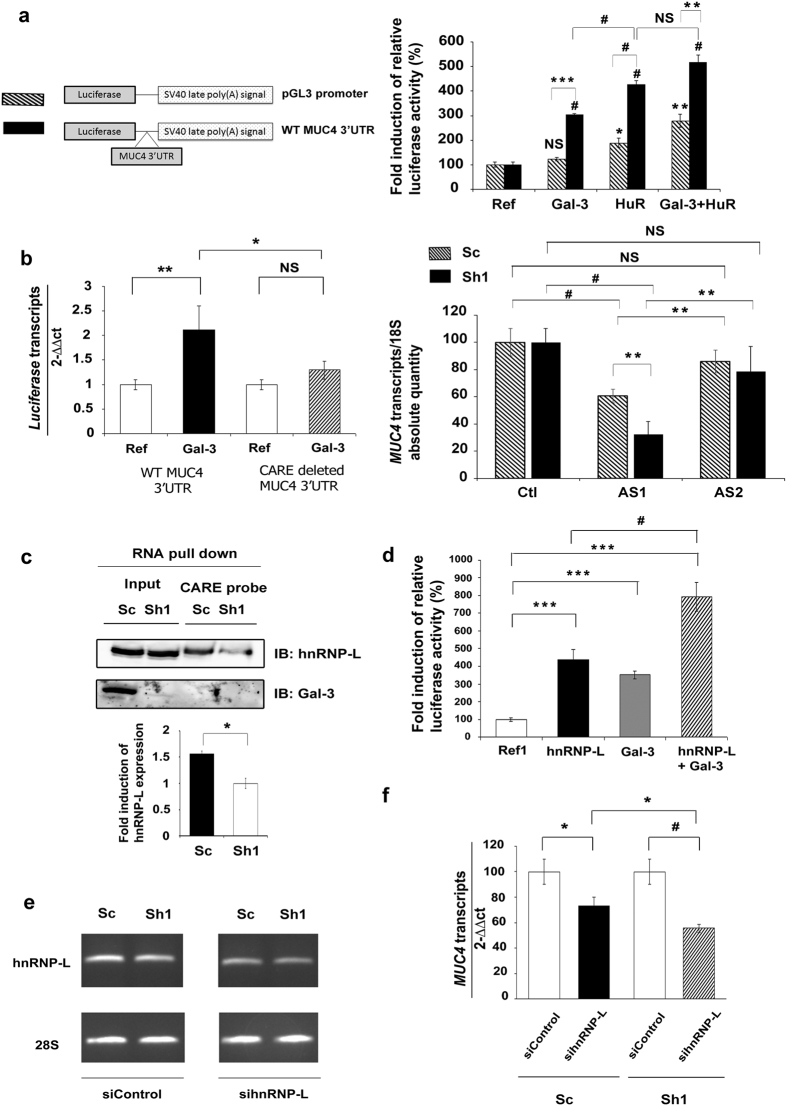
Mechanism of action of Galectin-3 on the 3′UTR of *MUC4* mRNA. (**a**) Left, schematic representation of pGL3 promoter or WT MUC4 3′UTR constructs. Right, Sh1 cells were transfected with the pGL3 promoter (hatched bars) or the *MUC4* 3′UTR luciferase reporter vector (black bars). pCMV6-XL4 Gal-3 and/or pCDNA3 HuR expression vectors were co-transfected. pSV-β-galactosidase expression vector was used as a co-transfection control. Luciferase activity was normalized to β-galactosidase activity. A 100% ratio was set for transfection performed with the corresponding reference. Results are presented as mean ± SD, n = 3. (**b**) Left panel: Sh1 cells were transfected with luciferase vector constructs containing wild-type or CARE-deleted *MUC4* 3′UTR, and co-transfected or not with pCMV6-XL4 gal-3 expression vector. *Luciferase* and *GAPDH* transcripts were quantified by RT-qPCR. Results are presented as mean ± SD, n = 3. Right panel: Sc cells (hatched bars) and Sh1 cells (black bars) were transfected with antisense oligonucleotides targeting either the CARE (AS1) or a region from the *MUC4* 3′UTR devoid of a regulatory element (AS2), or without oligonucleotide as a control (Ctl). *MUC4* and *18S* RNA were quantified by RT-qPCR. *MUC4* mRNA level present in “Ctl” was set arbitrarily at 100%. Results are presented as mean ± SD, n = 3. (**c**) Biotinylated RNA probe corresponding to the CARE of *MUC4* 3′UTR was incubated with Sh1 or Sc whole cell extracts (100 μg) and the interaction with hnRNP-L or Gal-3 was assessed by western blot. Input: 25 μg of whole cell extracts. Blots are representative of 2 independent experiments. Densitometric analysis of the bands was performed using ImageQuanTL software. (**d**) Sh1 cells were transfected as indicated in (**a**). pCMV6-XL4 gal-3, pCMV6-XL5 hnRNP-L expression vectors were used separately or combined. Results are presented as mean ± SD, n = 3. (**e**) Determination of siRNA activity by semi-quantification of *hnRNP*-*L* mRNA level by RT-PCR in treated cells with siRNA targeting hnRNP-L or non-targeting siRNA (siControl). (**f**) *MUC4* and *GAPDH* transcripts were quantified by qPCR after hnRNP-L inhibition by specific *si*RNA during 48 h. Results were expressed in comparison with siControl (arbitrarily set at 100%). Results are presented as mean ± SD, n = 3. *p < 0.05; **p < 0.02; ^#^p < 0.01; ***p < 0.001; NS, not significant by Student’s t-test.

**Figure 3:**
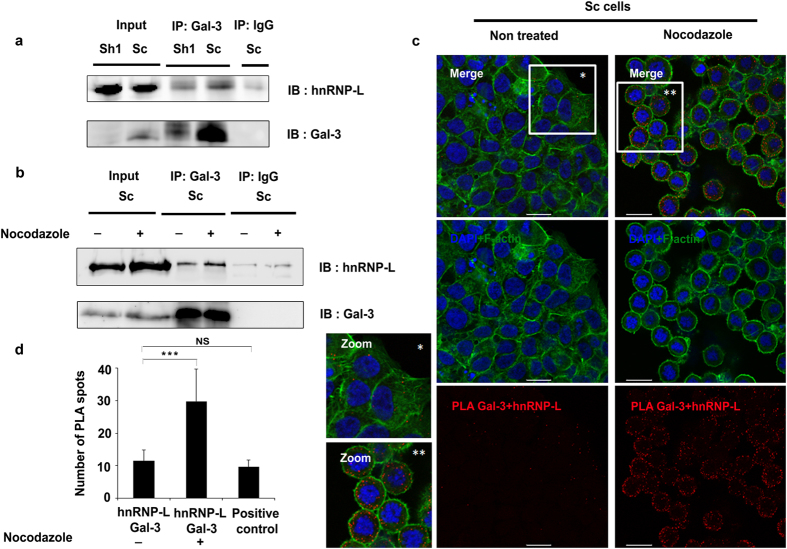
hnRNP-L interacts with Galectin-3. (**a**) Co-immunoprecipitation (co-IP) experiment was performed on 300 μg of total protein extracted from PFA crosslinked cells with 2 μg of anti-Galectin-3 antibody (Santa Cruz sc-20157) or isotype-matched antibody (Millipore, Normal rabbit IgG). Input: 25 μg. (**b**) Sc cells were treated with nocodazole (400 ng/ml, 16 h) and co-IP was performed as in (**a**). (**c**) Detection of Gal-3 and hnRNP-L interaction by proximity ligation assay (PLA) in Sc cells untreated or treated with nocodazole. The pictures show a single z-plane. PLA signals are shown in red, the nuclei are stained by DAPI (blue) and F-actin is stained with phalloidin (green). Merged images are shown in the upper panels. Scale bars = 20 μm. (**d**) PLA spots were manually counted with the ImageJ software using the Cell Counter plugin in at least 13 independent cells delimited with the F-actin staining. ***p < 0.001, NS, not significant by Student’s *t*-test.

We evaluated the ability of Gal-3 to stabilize *MUC4* mRNA through its 3′UTR using a luciferase-*MUC4* 3′UTR reporter construct and pGL3 promoter construct as a control. In transient co-transfection experiments, Gal-3 significantly increased the relative luciferase activity in the presence of *MUC4* 3′UTR, but had no effect in its absence (Fig. 2a). HuR, a classic RBP that recognizes AU-rich elements (AREs), significantly increased the luciferase activity produced by the pGL3 promoter construct (2-fold). The control plasmid contains six putative AREs in its endogenous 3′UTR. A higher induction by HuR (4-fold) was observed in presence of the *MUC4* 3′UTR which contains three additional AREs, two being canonical (ARE1 & 2) (Fig. S3) and one corresponding to a poly-U motif (ARE3)^23^. However, Gal-3 did not significantly increase the strong inductive effect of HuR on the 3′UTR of *MUC4* (Fig. 2a). By scanning the *MUC4* 3′UTR with the RBPDB tool^24^, we identified other putative *cis*-elements for RBPs such as KHSRP, AUF-1 and also a CA-repeat element (CARE) able to be recognized by hnRNP-L^25^, in the distal part of the 3′UTR (Fig. S3). We focused on the CARE motif, which is conserved in the 3′UTR of mouse and rat *Muc4* and absent from the 3′UTR of *MUC1* mRNA that is not stabilized by Gal-3. Deletion of the CARE motif from the luciferase-*MUC4* 3′UTR reporter construct abolished the ability of Gal-3 to increase luciferase mRNA levels (Fig. 2b, left panel). Moreover, antisense oligonucleotides (AS1) targeting the CARE motif significantly decreased the endogenous levels of *MUC4* mRNA, whereas non-targeting control antisense oligonucleotides (AS2) had no effect by comparison with untransfected control cells. Moreover, the decrease of *MUC4* mRNA levels was more pronounced in Sh1 than in Sc cells (Fig. 2b, right panel). Thus, Gal-3 exerts a stabilizing effect on *MUC4* mRNA through the CARE motif present in its 3′UTR. Since this element may serve as a platform for hnRNP-L binding^25^, we investigated the involvement of hnRNP-L in the stabilizing effect of Gal-3. Using RNA pull-down assays, we confirmed that hnRNP-L binds to the CARE motif present in the *MUC4* 3′UTR. hnRNP-L binding was significantly increased (mean: 1.6 fold) in Gal-3 expressing cells whereas hnRNP-L protein expression was not affected (Fig. 2c). Gal-3 itself did not bind to the CARE probe despite performing the reaction in a low salt buffer (60 mM NaCl) that favors its association to RNA^4^ (Fig. 2c). To address more directly whether Gal-3 stabilizing effect depends on hnRNP-L, we looked for an interplay between Gal-3 and hnRNP-L. In co-transfection experiments, Gal-3 significantly amplified the stimulatory effect of hnRNP-L on the *MUC4* 3′UTR (Fig. 2d). Moreover, the moderate siRNA-mediated knockdown of hnRNP-L (Fig. 2e) was sufficient to induce a significant reduction in endogenous *MUC4* mRNA levels that was greater in Gal-3-depleted cells than in control cells (Fig. 2f). Finally, we assessed the direct interaction between Gal-3 and hnRNP-L proteins using co-immunoprecipitation (co-IP) or proximity ligation assays (PLA) which facilitates protein complex detection. To stabilize weak interactions, cells were treated by formaldehyde before being immunoprecipitated with an anti-Gal-3 antibody. In these conditions and in agreement with recent data^26^, we observed an interaction between hnRNP-L and Gal-3 in whole Sc cell extracts (Fig. 3a). Treatment with nocodazole, a drug that triggers microtubule depolymerization and blocks the cells in the G2/M phase (Fig. S4a), revealed a more intense association of hnRNP-L to Gal-3 (Fig. 3b). Regarding the subcellular localization of the interaction, most PLA signals in basal state were observed in the perinuclear region although sparce distinct fluorescent spots were also found in the nucleus or the cytoplasm of Sc cells (Fig. 3c and S5, PLA controls in Fig. S6). The Gal-3-hnRNP-L interaction is relevant since the mean number of PLA spots (around 11 per cell, Fig. 3d) is equivalent to those obtained for a validated interaction such as MUC4-ErbB2 (around 9.6 spots/cell; Fig. 3d and S6). Interestingly, nocodazole treatment potentiated the interaction between Gal-3 and hnRNP-L in the cytoplasm of mitotic cells as demonstrated by a significant increase of the mean number of PLA spots (Fig. 3c,d) and also by the intensity of the co-localization assessed by immunofluorescence (IF) (Fig. S4b). In conclusion, Gal-3 interacts in the cytoplasm with hnRNP-L which mediates the stabilizing effect on *MUC4* mRNA through its interaction with the CARE motif present in *MUC4* 3′UTR. This interaction is increased during cell mitosis.

**Figure 4:**
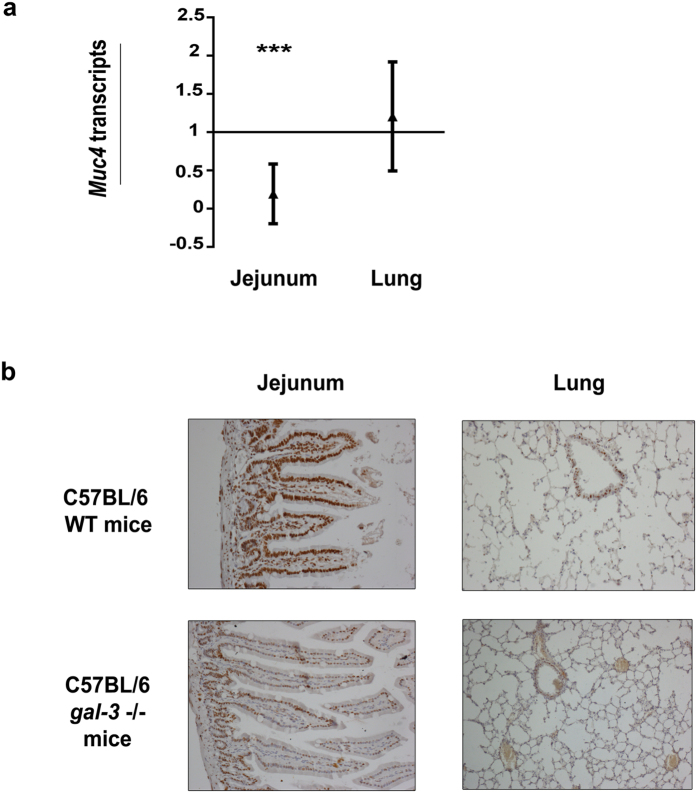
*Muc4* mRNA expression in Gal-3 null mutant (gal-3^−/−^) and wild type (WT) C57BL/6 mice. (**a**) Total mRNAs were extracted from jejunum (WT n = 9 (mice), gal-3^−/−^ n = 12) and lung (WT n = 14, gal-3^−/−^ n = 12). *Muc4* and *Gapdh* (internal control) transcripts were quantified by RT-qPCR using the 2^−ΔΔ^Ct method. *Muc4* mRNA levels was arbitrarily set at 1 in WT mice and results are expressed as fold variation in gal-3^−/−^ *vs* WT mice. ***p < 0.001; NS, not significant by Student’s *t*-test. (**b**) Analyses of hnRNP-L expression by immunohistochemistry in jejunum and lung tissues from WT and gal-3^−/−^ mice, n = 4 mice in each condition.

To address the physiological relevance of these results and taking advantage of the conservation of the CARE in the mouse *Muc4* 3′UTR, we used RT-qPCR to quantify *Muc4* mRNA levels in several epithelial tissues from wild-type (*wt*) and *gal*-*3*^−/−^ mice. *Muc4* mRNA levels were significantly lower in the jejunum of *gal3*^−/−^ mice compared with *wt* mice, while the levels were unchanged in lung (Fig. 4). In parallel, we evaluated hnRNP-L protein expression by immunohistochemistry. We observed that hnRNP-L was less expressed in the jejunum from *gal3*^−/−^ mice compared with *wt* mice whereas no obvious variation occurred for the lung (Fig. 4). The low hnRNP-L expression level likely contributed to the low levels of *Muc4* mRNA expression in *gal3*^−/−^ jejunum. All together, our results strongly support the hypothesis of a conserved and synergic interaction between Gal-3 and hnRNP-L to control *MUC4* mRNA stability both in normal and cancerous epithelial tissues.

### Galectin-3 is able to bind mature mRNAs in the cytoplasm

**Figure 5:**
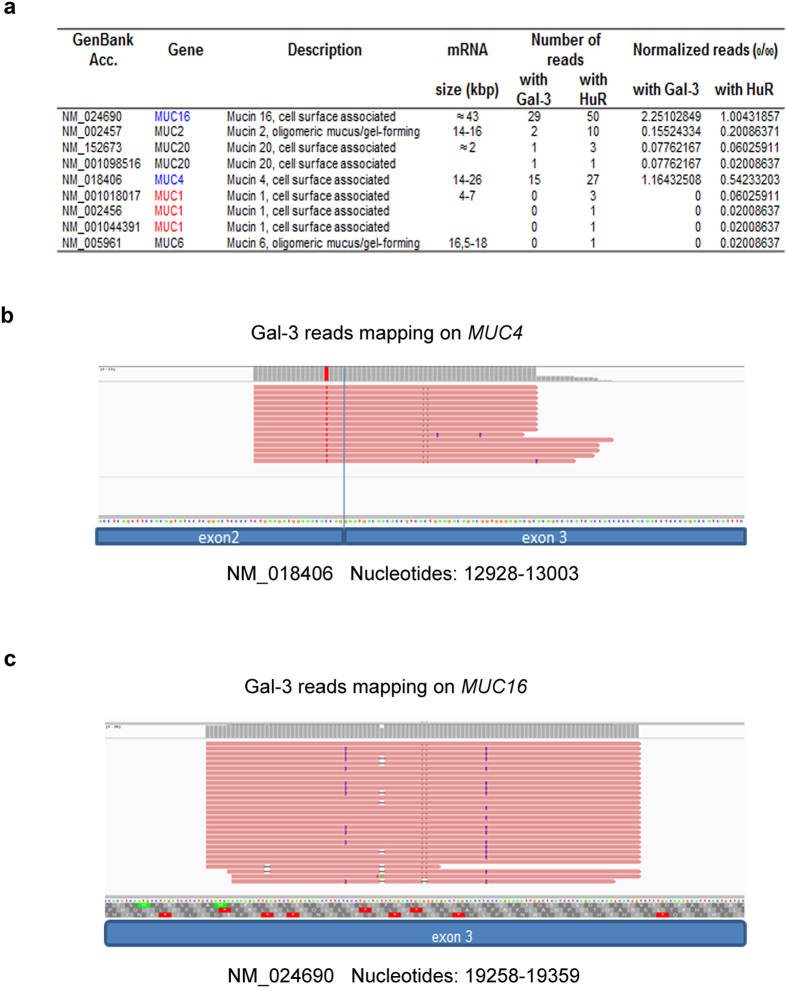
RNA-seq analysis of mRNAs immunoprecipitated by anti-Gal3 antibody. (**a**) The reads generated by RNA-Seq from anti-Gal-3 and anti-HuR RNA-IP were mapped against mucin transcripts using Bowtie2. Normalized reads were calculated by dividing the number of mapped reads by the total number of reads mapping on ENTREZ Gene x1000. (**b**) Distribution of RNA-Seq reads from anti-Gal-3 RNA-IP aligned on *MUC4* mRNA. Each read is indicated by a red bar. The longest read contains 75 bp. (**c**) Distribution of RNA-Seq reads from anti-Gal-3 RNA-IP aligned on *MUC16* mRNA. Each read is indicated by a red bar.

**Figure 6:**
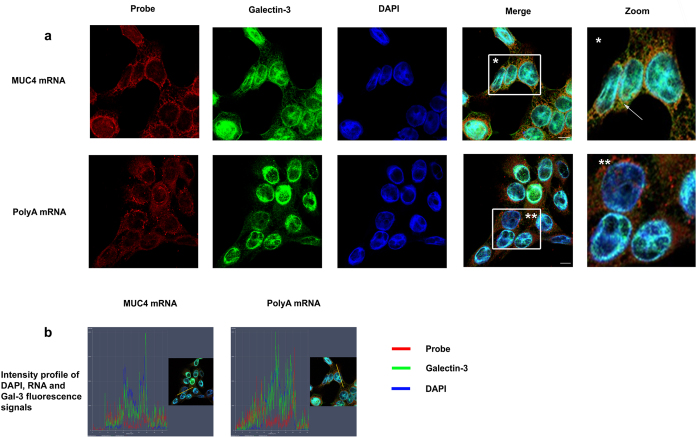
Galectin-3 interacts with mature mRNAs in the cytosol. (**a**) PolyA mRNA or *MUC4* specific transcripts were detected by FISH using either a Cy3.5 oligodT 40 mer or a Cy3.5 *MUC4* specific 48 mer probe directed against the tandem repeat (red) in Sc cells. Galectin-3 was detected by IF (green). Nuclei were stained by DAPI (blue). Analyses were performed using laser scanning confocal microscopy and the pictures show a single z-plane. Co-localization was assessed by merging the images (Merge) where yellow spots indicate co-localization of transcripts and Gal-3. A zoom is performed in the area delimitated by a white square and arrow highlights example of co-localization. Scale bar: 10 μm. (**b**) Fluorescence intensity profiles of Gal-3, DAPI and RNA probe staining in Sc cells. Fluorescence intensity was quantified across the cell along the yellow arrow (see inset) using the ZEN software (Zeiss microscope) and presented as a histogram (blue, DAPI; green, Gal-3; red, RNA probe). The profile displayed is representative of three carried out independently.

**Figure 7:**
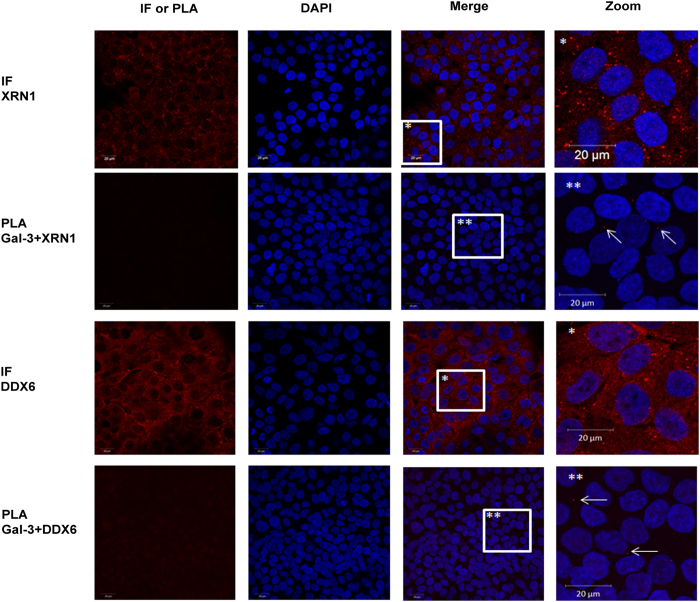
Galectin-3 interacts rarely with protein markers of P-bodies. Xrn1 and DDX6 were detected by IF (red) in basal conditions in Sc cells. Nuclei were stained with DAPI (blue). Detection of Gal-3 and Xrn-1 or DDX6 interaction was assessed by PLA. The pictures show a single z-plane. PLA signals are shown in red, nuclei were stained by DAPI (blue). A zoom is performed in the area delimitated by a white square and arrows highlight example of PLA spots. Scale bars = 20 μm.

Cells were treated with formaldehyde to preserve *in vivo* protein-protein and protein-RNA interactions^27^,^28^ and to avoid artefactual rearrangements of Gal-3-RNA complexes after cell lysis. Cross-linking was followed by RNA-IP with anti-Gal-3 or anti-HuR antibody followed by RNA-Seq analysis in Sc cells. HuR, a classic RBP that recognizes the AU-rich elements (ARE) in the 3′UTR^23^, was used as a positive control in the RNA-IP. As expected, HuR immunoprecipitated *MUC2* and *MUC4* mRNAs (Fig. 5a) that contains AREs in their 3′UTR together with *MUC16* that contains one ARE in at least one Genbank-referenced mRNA. Moreover, this analysis demonstrated that Gal-3 interacts with several mucin mRNAs such as *MUC4* and *MUC16* but not with those encoding *MUC1* (Fig. 5a). Concerning *MUC4* mRNA, all the RNA-Seq reads were aligned to the exon 2 exon 3 junction (Fig. 5b), thus demonstrating that Gal-3 interacts with a full-length mature spliced transcript at the end of the largest exon 2 (>12 kb) which encodes the tandem repeat (TR) region. All the reads for *MUC16* (Fig. 5c) aligned also with the largest exon (exon 3, estimated size >21 kb), which however does not encode the TR region. To determine the subcellular compartment(s) in which Gal-3 interacts with mucin mRNAs, whole poly-A or *MUC4* mRNAs were detected by fluorescence *in situ* hybridization (FISH) and Gal-3 was detected by IF. Merged images and fluorescence intensity profiles across single cells showed co-localization of *MUC4* mRNA with Gal-3 at the perinuclear region of Sc cells (Fig. 6, controls see Figs S7,S8). Thus, both RNA-IP and combined imaging data demonstrate that Gal-3 is able to bind mature mRNAs *in vivo* in the cytosol after their nuclear export. In the cytosol, dynamic mRNA exchanges take place between different granules that store untranslated mRNA in RNP complexes such as Stress Granules (SG) or P-bodies (PB) and the ribosomes where active translation occurs. To investigate whether Gal-3 may be part of SG or PB, we assessed Gal-3 interaction in the cytoplasm with several specific protein markers of SG and PB by PLA. Regarding PB, we selected GW182, Xrn1 and DDX6(P54) as protein markers and we did not observe a dot-like cytoplasmic staining using a commercial antibody against GW182 (not shown). However, numerous cytoplasmic foci were visible using an antibody which recognizes the 5′–3′ exoribonuclease Xrn1 or the ATP-dependent RNA helicase DDX6 (P54)(Fig. 7). Using PLA, we observed few fluorescent spots (typically 1 per cell) in the cell cytoplasm (Fig. 7 and S9,S10), indicating a rare interaction between Gal-3 and markers of PB.

**Figure 8:**
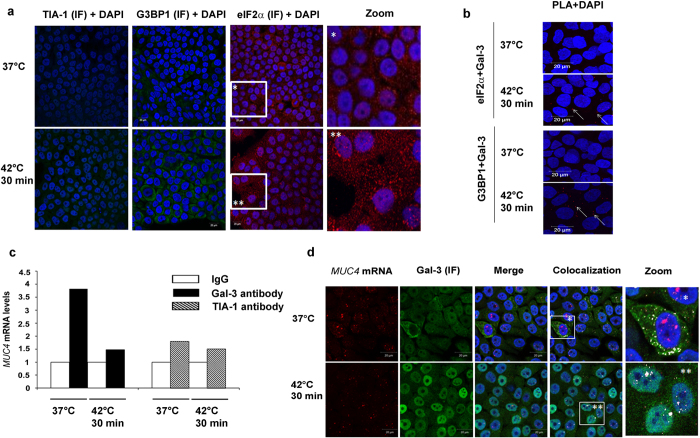
Galectin-3 interacts rarely with protein markers of SG which are not enriched in *MUC4* mRNA. (**a**) Localization of eIF2α (red), TIA-1 or G3BP (both in green) in Sc cells was assessed by IF in basal condition (37 °C) or after a heat stress (42 °C, 30 min) in Sc cells. Nuclei were stained with DAPI. Scale bars = 20 μm. Zooms were performed in the area delimited by a square. (**b**) Detection of Gal-3 and eIF2α or G3BP interaction assessed by PLA in basal condition (37 °C) or after a heat stress (42 °C, 30 min). PLA spots are shown in red and Nuclei were stained with DAPI. Arrows highlight example of PLA spots. Scale bars = 20 μm. (**c**) mRNAs-protein complexes were IP with 15 μg of anti-TIA1 (Santa Cruz sc-1751), anti-Gal-3 (Santa Cruz sc-2157) or corresponding isotype matched antibodies in Sc cells. *MUC4* mRNA were quantified by RT-qPCR. Results are expressed in comparison with *MUC4* mRNA IP with the corresponding IgG (arbitrarily set at 1); n = 1. (**d**) *MUC4* mRNAs were detected by FISH with a Cy3.5 *MUC4* specific 48 mer probe directed against the tandem repeat (red) in Sc cells. Gal-3 was detected by IF (green). Nuclei were stained by DAPI (blue). Co-localization was assessed by merging the images (Merge) and white points indicate co-localization of transcripts and protein of interest. Zooms are performed in the area delimitated by a white square. Experiments were performed in basal (37 °C) and stress conditions (cell incubation at 42 °C for 30 min). Scale bars: 20 μm.

Regarding SG, we selected TIA-1, eIF2α and G3BP1 as protein markers. The appearance of distinct cytoplasmic SG was observed after heat shock using the 2 latter markers, and especially eIF2α (Fig. 8a). We then looked whether Gal-3 interacts with G3BP1 and eIF2α by PLA approach. As shown in Fig. 8b and S11,S12, the number of PLA spots after heat shock remains low (typically1 to 3 per cell), especially if one considers the total number of SG (Fig. 8a). Therefore Gal-3 very rarely co-localizes with cytoplasmic SG after heat shock.

Next, we wanted to address whether *MUC4* mRNA might be associated with cytoplasmic SG which transiently store untranslated mRNAs. We performed a RNA-IP coupled with RT-qPCR to evaluate quantitatively the level of *MUC4* mRNA linked to Gal-3, or TIA-1 as a marker of SG, in basal or stressed conditions (Fig. 8c). In basal conditions (absence of SG), we observed an important *MUC4* mRNA enrichment in the anti-Gal-3 RNA-IP sample comparing with isotype-matched IgG control, in agreement with the imaging data showing a strong co-localization in the cell cytoplasm (Fig. 8d). This enrichment was reversed by a heat shock exposure which induces the formation of SG (Fig. 8a). Moreover *MUC4* mRNA was weakly enriched in the anti-TIA-1 RNA-IP both in basal and stressed conditions. All together, these data suggest that *MUC4* mRNA is associated with Gal-3 in cytoplasmic RNA-granules different from SGs.

## Discussion

Transcriptome data collected on nearly 1000 human cancer cell lines by the cancer cell line encyclopedia^29^, demonstrated that among the four tissues showing the highest expression levels of *MUC1, MUC4, MUC16* mRNAs, two tissues, bile duct and pancreas, also exhibited the highest levels of *LGALS3* transcripts. Using a more direct approach, we showed that Gal-3 depletion in our pancreatic cancer cell model is accompanied by a strong decrease of *MUC1* and *MUC4* transcript level(s) but with no significant change of *MUC16*. In addition in the human tissue levels, we highlighted a significant correlation only between *LGALS3* and *MUC4* mRNA levels suggesting that if a link exists between Gal-3 and mucin mRNA levels in cancer cells, it certainly concerns *MUC4* first. Therefore, the aim of this current work was to decipher how Gal-3 controls the expression of *MUC4* by addressing the two main mechanisms controlling mRNA levels i.e. transcription rate and transcript stability^30^. Our experiments showed no trans-activating effect of Gal-3 on *MUC4* promoter *in vitro*, but demonstrated that *MUC4* mRNA half-life dropped from 22.3 to 11.3 h in absence of Gal-3, whereas that of *MUC1* was not modified. To our knowledge, this is the first description of an mRNA stabilizing role for Gal-3 in the context of cancer cells.

Sharova *et al*.^31^, by studying a large set of mRNAs, demonstrated that stability depends on (i) the number of exon-junction by open reading frame (EJ/ORF) in a positive manner and (ii) the number and type of AU-rich elements (ARE) in the 3′UTR in a negative way. Since *MUC1* and *MUC4* transcripts do not differ in terms of EJ/ORF (1.23 vs 1.17) whereas their 3′UTR do, we asked whether the 3′UTR of *MUC4* was involved in the Gal-3 stabilizing effect. Our data suggest that its stabilizing effect depends on the CA Repeat Element (CARE) present in the human *MUC4* 3′UTR, which is conserved in rat and mouse *Muc4* homologs. This element acts as a binding platform for hnRNP-L, which is a RBP known to bind to CARE with high affinity and to promote the stability of *VEGF*^25^ or *CD154*^32^ mRNAs. We also showed that Gal-3 increases hnRNP-L binding to the CARE motif and potentiates its transactivating effect on *MUC4* 3′UTR while no direct binding of Gal-3 to the CARE motif was detected even after using experimental conditions that promote this interaction. We propose that hnRNP-L is the protein mediator of Gal-3 stabilizing effect on *MUC4* mRNA. This hypothesis is further supported by the absence of modification of *Muc4* expression in epithelial tissues of unchallenged *gal*-*3*^−/−^ mice that were not depleted of hnRNP-L protein.

The mRNA cycle in the cytosol consists of dynamic exchanges between various types of granules containing proteins and untranslated mRNAs (such as SG and PB) and the ribosomes where mRNAs are actively translated^33^. Therefore, the processes of mRNA decay, storage and translation are interconnected in eukaryotic cells and occur in competition with each other with the equilibrium between them determining the half-life of each mRNA species. In the present study, we showed that in the cytoplasm Gal-3 rarely co-localizes with Xrn-1 and DDX6, proteins constituent of P-bodies^34^, and TIA-1, eIF2α and G3BP1 which are hallmarks of SG^35^. In the same time, in basal conditions where SG do not exist, Gal-3 is strongly associated in the cell cytoplasm first with a mature form of *MUC4* mRNA and second with hnRNP-L. Recently, hnRNP-L was described as a component of IMP1-containing RNP granules which store untranslated mRNAs^36^ but its role as an actor or a passive bystander was not elucidated^36^. We thus propose that *MUC4* mRNAs are stored and protected in cytoplasmic RNA granules containing both Gal-3 and hnRNP-L in basal conditions.

In mitotic cells we observed a stronger interaction between hnRNP-L and Gal-3. Interestingly, mitotic cells are characterized by an absence of SG^37^ and a stalling of ribosomes on the mRNAs which are not translated and protected. We therefore suggest that Gal-3 and hnRNP-L may play a broad role on the cytoplasmic mRNA homeostasis especially during cell mitosis.

Intriguingly, Gal-3 is able to stabilize *MUC4* but not *MUC1* mRNA, in our pancreatic cancer cell model. However, exocrine pancreatic cells express the membrane-bound MUC1 both physiologically and in all stages of carcinogenesis, while MUC4, which is absent from normal adult exocrine pancreatic cells, becomes expressed in the ducts, which are the setting of inflammatory or cancerous lesions. Therefore, we propose that *MUC1* constitutes a kind of epithelial “housekeeping” mucin gene expressed by most epithelial cells, whereas in the pancreas, *MUC4* belongs to a set of genes induced in response to stress and injury^19^ and is able to modulate the inflammatory response^38^ as Gal-3 does^10^. Our hypothesis is that *MUC4* mRNA requires a specific control of its trafficking and/or decay by Gal-3 in this context.

Previous studies already showed that despite the absence of a RNA recognition motif, Gal-3 interacts with pre-mRNA at the 5′end of the introns via U1 snRNP in the nucleus where it promotes their splicing^3^,^26^. We showed by RNA-Seq analysis that Gal-3 binds to a mature mRNA after removal of introns and export in the cytosol. Our data, together with those reported by others based on mass spectrometry approaches^39^, and different cell models, demonstrate that Gal-3 is a non-classical RBP and thus contribute to expand the repertoire of RBPs^40^. The experimental procedures used in the present study, which promote cross-linking between proteins and mRNAs, as well as between protein partners, indicate that Gal-3 binds mature mRNAs via interactions with classical RBPs since Gal-3 basically lacks an RRM, in accordance with previous reports^5^. Two hypothesis, at least, could be proposed to explain where and how Gal-3 binds to mature mRNAs. In the first hypothesis, Gal-3 is loaded onto pre-mRNA as part of the U1 snRNP^5^, remains associated with mature mRNA and shuttles with it from the nucleus to the cytosol^26^. In this case, Gal-3 would function as a tag reminiscent of its role in constitutive splicing by analogy with the proteins of the exon junction complex (EJC)^41^. In the alternative more dynamic hypothesis, Gal-3 in the nucleus dissociates from pre-mRNAs after splicing due to the removal of the U1 snRNP, and re-associates in the cytosol with a subgroup of mature mRNAs after nuclear export by interacting with a network of RBPs, including hnRNP-L, in order to perform its new function i.e. regulation of mRNA metabolism. Gal-3 can also shuttle back in the nucleus in response to heat shock (Fig. 8) or oxidative stress^42^, a change that probably contributes to gene reprogramming in response to stress^43^.

In conclusion, our data clearly expand the function of Gal-3 in mRNA metabolism by showing for the first time a post-transcriptional role for Gal-3 in the stabilization of mature *MUC4* mRNAs in the cytoplasm. Further work is required to demonstrate whether Gal-3 regulates the half-life of other transcripts.

## Materials and Methods

### Cell culture and transfections

Control Sc cells and Gal-3 knock-down Sh1 cells (derived from CAPAN-1 pancreatic cancer cells) were obtained and cultured as previously described^20^. Nocodazole (400 ng/ml; 16 h) was used to synchronize the cells in the G2/M phase. The details of transfection and siRNA silencing are provided in Supplementary Information (SI).

### Murine epithelial samples

Wild type and Gal-3 null mutant (*gal*-*3*^−/−^) mice were of the C57BL/6 background. All methods were carried out in accordance with the relevant guidelines and regulations (protocols approved by the Comité d’éthique Buffon from Institut Jacques Monod under the approval number A75-13-17). Tissues (jejunum and lung) were sampled from adult mice and were immediately snap-frozen in liquid N2 for RNA extraction or embedded in paraffin for immunohistochemistry.

### Human samples from tumorotheque

Ten tumour samples were obtained from patients with a pancreatic adenocarcinoma. Informed consent was obtained from all subjects. All methods were carried out in accordance with the relevant guidelines and regulations (protocol approved by the Tumorothèque de Lille under the approval number CSTMT220). Immediately after surgical resection, 2 small parts of the tumour sample were snap-frozen in liquid N2 after morphological control and stored at −80 °C in the Tumorothèque of C2RC (Centre de Référence Régional en Cancérologie) Lille; one part was representative of normal pancreas and the other of adenocarcinoma. The rest of the tumour sample was fixed and embedded in paraffin for routine pathological examination.

### Reversion experiment and Gal-3 Inhibitor treatment

Cells were cultured in 24-wells plates in serum free medium for 6 hours before adding 4 μM of recombinant Gal-3. After treatment, RNA extraction and cDNA synthesis were performed with the “Superscript III Cells Direct cDNA Synthesis System” (Life Technologies). Semi-quantitative PCR was carried out as previously described^44^,^45^.

For Gal-3 inhibitor treatment, confluent cells were treated with the inhibitor or controls for 6 h (200 μM). mRNA were then extracted as previously described^20^ and quantified by RT-qPCR.

Details of the synthesis of r-Gal-3, Gal-3 inhibitor and control sugar are provided in SI.

### RT-PCR

Semi quantitative RT-PCR was carried out as previously described for *MUC4*^44^ and with the following primers for *18S*: 18SF: 5′ GGACCAGAGCGAAAGCATTTGCC 3′ and 18S R: 5′ TCAATCTCGGGTGGCTGAACGC 3′.

### qPCR

#### Murine epithelial samples

Tissue samples were disrupted before performing RNA extraction and cDNAs were prepared as previously described^20^. *Muc4* transcripts from mice were quantified by qPCR (relative quantification, SsoFast Evagreen Supermix kit (Biorad)) using the 2^−ΔΔ^Ct method and *Gapdh* as an internal standard. Each sample was run in triplicate. Primers used are described in SI.

#### Cell lines and human samples from tumorotheque

Total RNA from the ten sample pairs was extracted using “Nucleospin RNA II” from Macherey Nagel. RNA concentration and purity were determined with Nanodrop. Total RNA from cell lines was extracted as previously described^20^. *MUC1, MUC4, MUC16, LGALS3* and *18S* were quantified by RT-qPCR (absolute quantification, Taqman technology) using *18S* as an internal control^20^. Each sample was run in triplicate. Primers are available in SI.

In cell lines, quantification of *MUC4* after siRNA transfection and mRNA extraction was carried out by RT-qPCR (relative quantification, Taqman technology) using the 2^−ΔΔ^Ct method and *GAPDH* as an internal standard. In cells transfected with pGL3 vectors, *Luciferase* mRNA were quantified by RT-qPCR (relative quantification, SsoFast Evagreen Supermix kit (Biorad)) using the 2^−ΔΔ^Ct method and human *GAPDH* as an internal standard. Each sample was run in triplicate. Primers are available in SI.

### mRNA half-life

Sc and Sh1 cells were treated in presence of 8 μg/ml actinomycin D (Act. D) as previously described^18^. Samples were collected 3, 6, 9, 12, 24 and 30 h after addition of Act. D due to the expected long half-life of mucin transcripts^18^. Total RNA and cDNAs were prepared as described^20^. Absolute quantification of *MUC4* transcripts was carried out by qPCR.

### Western Blot

Western-Blot were performed as previously described^20^,^46^. Membranes were incubated overnight at 4 °C with the following antibodies: hnRNPL (Abcam ab6106, 1/2000), Galectin-3 (Abcam ab31707, 1/300) and β-actin (Sigma AC-15, 1/5000). The membranes were then incubated with peroxydase-conjugated secondary antibodies (Sigma-Aldrich) and revelation was performed with LAS 4000 (Fujifilm) using the West Pico chemoluminescent substrate (Perbio).

### RNA Immunoprecipitation (RNA-IP) and RNA sequencing (RNA-Seq)

Sc cells were cultured in 10 cm diameter culture dishes, and fixed in 1% (v/v) formaldehyde during 10 min to covalently crosslinking proteins to nucleic acids *in vivo*. RNA-IP was then performed as described^47^ with the following modifications: protease inhibitor cocktail, vanadyl ribonucleoside complexes solution and RNasin RNase inhibitor were added in the RIPA buffer. Fixed cells were harvested, re-suspended in RIPA buffer and sonicated (two rounds of five minutes, Bioruptor). The preclearing step (with 75 μl of EZview™ Red Protein A Affinity Gel) and the immunoprecipitation (with 15 μg of antibody directed against galectin-3 (Santa Cruz, sc-20157), HuR (Santa Cruz, sc-20694), TIA-1 (Santa Cruz, sc-1751) or with isotype matched at 4 °C) were carried out as described^48^. Beads were washed five times with RIPA buffer containing either 1 M of urea (galectin-3) or 3 M of urea (HuR, TIA-1). After RNA extraction by acid phenol-chloroform and ethanol precipitation in presence of GlycoBlue (Life Technologies), samples were treated with DNase I for 10 min and purified RNAs were stored at −80 °C. For *MUC4* qPCR, mRNA were retrotranscribed and quantified by qPCR as described in previous section. For RNA-Seq, purified RNAs from two dishes were pooled before RNA-Seq. RNA-Seq was performed on a PGM System (Life Technologies) using the Ion Total RNA Seq Kit V2 without fragmentation by RNAse III. The yield and size distribution were evaluated after amplification-purification of the cDNA. A second round of purification was carried out to eliminate primer dimers using Agencourt AMPure XP beads. Sequence reads were analyzed with Torrent Server 3.0 for barcode analysis and adaptor trimming. The reads were aligned with bowtie2^49^ on refSeq mRNA downloaded from UCSC. Then we only take into account reads mapping on mRNA with an ENTREZ Gene ID using ID converter^50^.

### FISH and imaging studies

Sc and Sh1 cells were grown on Lab-Tek Chamber slides (Nunc) until 50% confluency before being fixed in 4% paraformaldehyde and permeabilized with 0.2% saponin. Then, hybridization was carried out as previously described^51^, except that Triton X100 was replaced by saponin, using either an oligo-dT Cy3.5 tagged probe (1 h at 37 °C) or a *MUC4* specific Cy3.5 tagged probe (4 h at 42 °C) (Eurogentec) directed against the tandem repeat^52^. For additional immunofluorescence, an antibody directed against Gal-3 (Abcam ab31707, 1/150), GW182 (Abcam ab70522, 1/200) TIA-1 (Santa Cruz sc1751, 1/200), Xrn-1 (Abcam 70259), eIF2α (Enzo, ADI KAP C130D), DDX6 (Bethyl A300-461A) and/or G3BP1 (BD 611126) was used. AlexaFluor^®^ 488 chicken anti-rabbit, AlexaFluor^®^ 488 goat anti-mouse and AlexaFluor^®^ 633 chicken anti-goat (Invitrogen) were used as secondary antibodies (dilution 1/500) according to the origin of the primary antibody. Nuclei were stained with DAPI. The confocal observations were performed with an Inverted laser scanning Axio observer microscope LSM 710 (Carl Zeiss) and EC PLAN Apochromat 63x/1.4 NA or 40x. Acquisitions were performed in sequential mode and analyzed with the Zeiss Efficient Navigation software (ZEN, Carl Zeiss). Stress conditions were performed by incubating cells for 30 min at 42 °C.

### Proximity ligation assay

For Proximity ligation assays, cells were grown on Lab-Tek chamber slides (Nunc), fixed with 4% paraformaldehyde (PFA), and permeabilized with 0.2% saponin (w/v, Sigma) and satured with 3% Bovine Serum Albumin (BSA, w/v, Sigma) and 0.2% saponin. Cells were incubated overnight at room temperature (RT) with an anti-Galectin 3 antibody (above) and either anti-hnRNP-L (Abcam ab6106, 1/200), anti-Xrn-1, anti-eF2α, anti-DDX6, or anti-G3B1 (above) antibodies. Then, Lab-Tek chambers were washed three times (D-PBS + Mg^++^ Ca^++^) before proceeding with the proximity ligation assay using Duolink^®^ *In Situ* reagents (Olink^®^ Bioscience) as described by the manufacturer’s instructions. F-Actin were stained with phalloidin-FITC (Life technologies). Negative controls (absence of primary antibodies, proteins with different subcellular localization) and positive controls (known protein partners) were systematically performed (Fig. S5). Slides were then mounted in mounting medium containing diamidino-2-phenylindole (DAPI) and visualized with a Zeiss LSM 710 confocal microscope (Carl Zeiss Microscopy); images were captured and analyzed with the Zeiss Efficient Navigation software (ZEN, Carl Zeiss).

### RNA Pull Down

Biotinylated RNA probe corresponding to CARE region of *MUC4* 3′UTR (Fig. S3) was incubated with Sh1 or Sc whole cell extracts (100 μg) as described^53^. Incubation and washes were carried out with buffer containing 60 mM NaCl to prevent the dissociation of Gal-3 from the RNA. The pull down material was analysed by western blot.

### Immunohistochemistry

hnRNP-L immunohistochemistry was performed as previously described^54^ with the following modifications: saturation was performed by incubation in mouse Ig blocking reagent (1 h, RT, kit MOM PK2200, Vector). Then, sections were incubated 1 h using anti-hnRNP-L antibody at RT (mouse, Abcam ab6106, 1/100) in M.O.M diluent + Triton X 100 0.1% followed by an incubation for 30 min with M.O.M biotinylated anti-Mouse IgG reagent and for 5 min with M.O.M Vectastain Elite solution.

### Co-immunoprecipitation

Co-IP was performed as previously described^20^ with an anti galectin-3 antibody (Santa-Cruz sc-20157) and isotype-matched antibody (Millipore Normal Rabbit) with a preliminary step of formaldehyde crosslinking^55^. Co-IP proteins were analyzed by western blotting.

### Statistical analyses

Statistical analyses were performed using Student’s *t*-test and were considered significant for the following P-values at least <0.05.

## Additional Information

**How to cite this article**: Coppin, L. *et al*. Galectin-3 is a non-classic RNA binding protein that stabilizes the mucin *MUC4* mRNA in the cytoplasm of cancer cells. *Sci. Rep.* **7**, 43927; doi: 10.1038/srep43927 (2017).

**Publisher's note:** Springer Nature remains neutral with regard to jurisdictional claims in published maps and institutional affiliations.

## Supplementary Material

Supplementary Information
