# Supplementary material for: Galectin-3 is a non-classic RNA binding protein that stabilizes the mucin MUC4 mRNA in the cytoplasm of cancer cells
Source: Sci Rep. 2017 Mar 6;7:43927. doi: 10.1038/srep43927 (PMC5338267; doi:10.1038/srep43927)
Supplement: Supplementary Information [file srep43927-s1.pdf]

Galectin-3 is a non-classic RNA binding protein that stabilizes the mucin *MUC4* mRNA in the cytoplasm of cancer cells

Lucie Coppin, Audrey Vincent, Frédéric Frénois, Belinda Duchêne, Fatima Lahdaoui, Laurence Stechly, Florence Renaud, Céline Villenet, Isabelle van Seuningen, Emmanuelle Leteurtre, Johann Dion ,Cyrille Grandjean, Françoise Poirier, Martin Figeac, Delphine Delacour, Nicole Porchet & Pascal Pigny

Supplementary Information

Supplementary Methods

Synthesis of Recombinant Gal-3, Gal-3 inhibitor and control sugar

LGALS3 (NM_002306) human full cDNA clone corresponding to Gal-3 1-251 amino-acids was subcloned into pET15b vector with a 6xHis tag fused at the N-terminus (Merck Chem.) and the resulting recombinant plasmid was used to transform competent *E. coli* BL21 pLysS (DE3) (Merck Chem.). Supernatant containing Gal-3 was loaded on a Ni-NTA affinity resin (Sigma-Aldrich) and eluted in a Tris buffer with 250 mM imidazole. The fractions containing Gal-3 were pooled, dialysed against 1X Phosphate Buffer Saline, and concentrated using a vivaspin concentrator (Sartorius). Synthesis of 4-O-[3-O-(3-methoxybenzyl)-β-D-galactopyranosyl)]-1-azido-1,2-dideoxy-2-(3-methoxybenzoyl)-β-D-glucopyranose (Gal-3 Pos) and of 1,2;4',6'-di-O-(4-methoxybenzylidene-1-α-D-cellobiopyranoside (Gal-3 Neg) follows previously described procedure[1]. The former compound, a di-aromatic derivative of the lactosamine, is an inhibitor of Gal-3 CRD (Kd=0.77 μM as determined by isothermal microcalorimetry). The latter derivative is a di-aromatic disaccharide derived from cellobiose where the two sugar subunits are β(1→4) linked as in Gal-3 Pos. However Gal-3 Neg features glucose instead of galactose moiety, further protected at both 4- and 6-hydroxy positions. Therefore Gal-3 Neg does not bind to Gal-3 as it lacks structural elements mandatory for recognition of the CRD.

qPCR primers

Primers and probes sequences for mRNA quantification in mice by EvaGreen® were as follows: *mMuc4* F: 5'CCTTCACTGATAACCGCTGCTT 3', *mMuc4* R: 5'GCGGAGGCATTTTCATCC 3'; *mGapdh* F: 5' AGGTCGGTGTGAACGGATTTG 3', *mGapdh* R: 5' TGTAGACCATGTAGTTGAGGTCA 3'.

Primers and probes sequences for mRNA quantification in human tissues, Sc and Sh1 cells by TaqMan® were as follows : *MUC4* F: 5' TCAGCTGAGGCCTTGCCTT 3', *MUC4* R: 5' TCAGTCACCTTCCCTTTTCCA 3', *MUC4* probe: 5' FAM-TAAGGCGCCATTGCTTTTGGGAGA-Tamra 3' *;* *MUC1*F: 5' CAGACGTCAGCGTGAGTGATG 3', *MUC1* R: 5' CTGACAGACAGCCAAGGCAAT 3', *MUC1* probe : 5' FAM-TGCTGGTCTGTGTTCTGGTTGCGCT-Tamra 3' ; *MUC16* F: 5' CAGTCAACTACATGACACATT 3', *MUC16* R: 5' ACTCTGTCATCTCTCCGAGCC 3', *MUC16* probe 5' FAM-GTGATGGTGAAATTCAGGTAGA-Tamra 3' ; *LGALS3* F: 5'GCCACTGGCCCCTATGG 3', *LGALS3* R: 5' CCCAGGCAAAGGCAGGTTATA 3', *LGALS3* Probe: 5'FAM-CCACTGATTGTGCCT-Tamra 3'; *GAPDH* F: 5' CCACATCGCTCAGACACCAT 3', *GAPDH* R: 5' CCAGGCGCCCAATAC 3', *GAPDH* Probe: 5' VIC-AAGGTGAAGGTCGGAGTCAACGGATTTG-Tamra 3'. 18S ribosomal RNA was detected using Ribosomal RNA Control Reagents (Applied Biosystems).

Primers sequences for *luciferase* mRNA quantification in Sh1 cells by EvaGreen® were as follows *luciferase* F: 5' GCCTGAAGTCTCTGATTAAGT 3', *luciferase* R: 5' CCAGGCGCCCAATACG 3'; *GAPDH F*: 5' CCACATCGCTCAGACACCAT 3', *GAPDH* R: 5' CCAGGCGCCCAATACG 3'.

Transient transfections with expression vectors and luciferase assay

Co-transfections experiments in Sh1 cells were performed using Effectene reagent (Qiagen) in the presence of 1 µg of each *MUC4* (a)[2]- or *MUC1*-(gift of Gendler SJ) promoter deletion constructs cloned in pGL3 luciferase vector and 0.2 µg pCMV6-XL4 Gal-3 expression vector (Origene) or empty pCMV6-XL4 vector. Total protein content in the extract (4 µl) was measured using the bicinchoninic acid method in 96-well plates (Pierce, Thermo Fisher Scientific) and used for normalization.

The 3'UTR of *MUC4* (493pb) was cloned into the luciferase pGL3 promoter vector (Promega)[3]. Sh1 cells were cotransfected with 1 µg of the *MUC4* 3'UTR-pGL3 vector or corresponding empty vector alone (references), and with 0.2 µg of an expression vector encoding either galectin-3 (pCMV6-XL4), HuR (pcDNA3, kind gift of J.A. Steitz, Yale University), hnRNP-L (pCMV6-XL5, Origene) using Effectene reagent (Qiagen) as previously described[4]. Quantification of luciferase activity was performed as previously described[5]. Transfection efficiency was checked by performing co-transfection with 0.2 µg of pSV-β-Galactosidase (Promega) and by measuring β-Galactosidase activity[6]. The relative luciferase activity is calculated as the ratio of luciferase activity/β-galactosidase activity. For each experiment results are expressed as fold induction of the corresponding references.

Deletion of the CA repeat element of *MUC4* 3'UTR was carried out using the QuikChange Site-directed mutagenesis kit (Stratagene) according to the manufacturer instruction and using the following primers : Forward: 5' TGTGCACGCGCGCGTGGAGTTCATAATGTGGTGATGG 3' and reverse: 5' CCATCACCACATTATGAACTCCACGCGCGCGTGCACA 3'.

Transfections with siRNA

Transient inhibition of hnRNP-L was performed as previously described[5] with DharmaFECT 2 from Dharmacon (GE Healthcare) using a pool of siRNA (final concentration 25 nM, Dharmacon). The hnRNP-L target sequences present in the pool were: GCGAUGAGCUGGGAGUGAA, UGAAAGUAUUCUCAGGCAA, GUUUGUAGAGGCUUACUUA, UAAGAUGAACUGUGACCGA. Controls were performed using a non-targeting siRNA (siControl) from Dharmacon composed of siARN with the following sequences: UGGUUUACAUGUCGACUAA, UGGUUUACAUGUUGUGUGA, UGGUUUACAUGUUUUCUGA, UGGUUUACAUGUUUUCCUA. 48 hours after transfection, mRNA were extracted and quantified by RT-qPCR. Preliminary experiments were carried out to check the efficiency *of hnRNP-L* silencing by semi-quantitative PCR (hnRNPL F: 5' TTCTGCTTATATGGCAATGTGG 3', hnRNPL R: 5' GACTGACCAGGCATGATGG 3').

Transfections with Antisense oligonucleotides

Antisense oligonucleotides were designed to target the CARE region of *MUC4* 3'UTR (AS1: 5' TCGTGTGTGTGTGTGTGTGTGTGCAC 3') or a region from the 3'UTR of *MUC4* devoid of a regulatory element (AS2: 5' CACAGGCTAGTGTCCTTCTGTGGGT 3'). Sc and Sh1 cells were transfected by AS oligonucleotides (final concentration 1 µM) using Oligofectamine (Life technologies, Thermo Fisher Scientific) according to the manufacturer's instruction. 48 hours after transfection mRNAs were extracted and *MUC4* and *18S* were quantified by RT-qPCR.

Flow cytometry

Cell cycle was studied as previously described[7]. Briefly, cells were fixed in ethanol, stained with propidium iodide and sorted into 2N and 4N population by flow cytometry. Analyses were performed on the Cyan ADP analyzer (Beckman Coulter).

Antibodies used for PLA positive and negative controls

The following antibodies were used for PLA controls : anti-MUC4 (Santa Cruz, 8G7, 1/200), ZEB-1(Cell Signalling 3396, 1/200) and anti-ErbB2 (Santa Cruz, C18, 1/200).

1. André, S. *et al.* Combining carbohydrate substitutions at bioinspired positions with multivalent presentation towards optimising lectin inhibitors: case study with calixarenes. *Chem. Commun.* **47**, 6126-6128 (2011).

2. Perrais, M. *et al.* Characterization of human mucin gene MUC4 promoter: importance of growth factors and proinflammatory cytokines for its regulation in pancreatic cancer cells. *J. Biol. Chem*. **276**, 30923-30933 (2001).

3. Lahdaoui, F. *et al*. miR-219-1-3p is a negative regulator of the mucin MUC4 expression and is a tumor suppressor in pancreatic cancer. *Oncogene* **34**, 780-788 (2015).

4. Fauquette, V. *et al.* Transcription factor AP2–α represses both the mucin MUC4 expression and pancreatic cancer cell proliferation. *Carcinogenesis* **28**, 2305-2312 (2007).

5. Skrypek, N. *et al.* The MUC4 mucin mediates gemcitabine resistance of human pancreatic cancer cells via the Concentrative Nucleoside Transporter family. *Oncogene* **32**, 1714-1723 (2013).

6. Van Seuningen, I., Perrais, M., Pigny, P., Porchet, N. & Aubert, J. P. Sequence of the 5'-flanking region and promoter activity of the human mucin gene MUC5B in different phenotypes of colon cancer cells. *Biochem. J.* **348**, 675-686 (2000).

7. Corvaisier M, Bauzone M, Corfiotti F, Renaud F, El Amrani M, Monté D, Truant S, Leteurtre E, Formstecher P, Van Seuningen I, Gespach C, Huet G.Regulation of cellular quiescence by YAP/TAZ and Cyclin E1 in colon cancer cells: Implication in chemoresistance and cancer relapse. *Oncotarget*. (2016). [Epub ahead of print]

Supplementary legends

Figure S1. Transcriptional regulation of *MUC1* and *MUC4* promoters by Galectin-3*.*

Sh1 cells were seeded at 0.4x10^6 cells per well in 6-well plates. Co-transfections experiments were performed the next day using Effectene reagent (Qiagen) in the presence of *MUC4* **(a)**- or *MUC1*-**(b)** promoter deletion constructs cloned in pGL3 luciferase vector and Gal-3 expression vector or empty pCMV6 vector. Total cell extracts were prepared after a 48 h incubation at 37°C using 1X RLB buffer (Promega). Luciferase activity was measured on a Mithras luminometer on 20 µl of cell extracts using luciferase assay reagent (Promega). Dosage of total proteins was used for normalization. Results are expressed as fold induction of luciferase activity relative to the empty vector (arbitrarily set to 1). Values are means of three independent experiments were co-transfections were run in triplicate. The

error bar represents the SD from the mean. p-values were determined by student's t-test; NS, not significant.

Figure S2. *MUC16, MUC1* and *LGALS3* expression in human PDAC tissues

Total RNAs were extracted from human PDAC tissues and surrounding healthy pancreatic tissues (10 couples of tissues), and *MUC1, MUC16, LGALS3* and 18S RNA were quantified by absolute RT-qPCR. Results are expressed as an induction factor of *MUC1* or *MUC16* and *LGALS3* expression in tumor tissues in comparison with corresponding healthy pancreatic tissues (mRNA level arbitrarily set at 1). No correlation is observed between *LGALS3* expression and *MUC1* (r^2=0.0022, NS*)* or *MUC16* (r^2=0.0701, NS*)* expression in human PDAC tissues. Groupe A corresponds to patients with low levels of *MUC4* and *LGALS3*, groupe B to patients with high levels of *MUC4* and *LGALS3*. NS: Not Significant.

Figure S3. Schematic representation of the 3'UTR of *MUC4* encoded by exon 25 showing the end of exon 24, intron 24 and exon 25 of the gene.

Are depicted on the figure:

- The stop codon is in bold characters;
- The CARE element is shown in pink characters;
- The first and last nucleotide of the CARE probe are shown in red bold framed characters;
- Regions targeted by AS1 and AS2 antisense oligonucleotides are underlined;
- AU-rich elements are depicted in blue;
- Putative site for miRNA are depicted in purple;
- The polyadenylation site is in red.

The *cis*-binding elements were identified using RBPDB tool.

The whole 3'UTR is conserved across MUC4 splicing variants sv0 (full length), sv1 to sv8, MUC4/X and MUC4/Y (BLAST analysis run on Non-RefSeq RNA database (286,496 sequences)).

Figure S4. Effect of nocodazole treatment on hnRNP-L and Gal-3 association

(a) Assessment of nocodazole treatment efficiency. Graph displays cell cycle analysis of Sc cell treated (red line) or not (blue line) with nocodazole (400 ng/ml, 16h) by flow cytometry. Profiles show that cells accumulate in G2/M after nocodazole treatment.

(b) Gal-3 (red) and hnRNP-L (purple) were detected by IF in Sc cells after nocodazole treatment. Nuclei were stained with DAPI and F-actin with phalloidin. Co-localization was assessed with images merging (merge) and shown with white spots. Zooms are performed in the area delimited by a white square. Scale bars: 20 µm.

Figure S5. PLA hnRNP-L+Gal-3

Grey scale image of the PLA interaction described in Figure 3c.

Figure S6. PLA controls

(a) Specificity controls of PLA in Sc cells were performed without primary antibodies but with anti-mouse MINUS probe or anti-goat MINUS probe and anti-rabbit PLUS probe. The nuclei are stained with DAPI (blue). PLA spots were counted in each cells to determine an average spot number per cell (see Figure 3d).

(b) Control of PLA sensitivity. Upper panel (positive control): example of a PLA staining obtained for a validated protein-protein interaction (MUC4 and ErbB2). On the merge, numerous red spots can be observed in several cells. Lower panel (negative control): primary antibodies directed against two proteins which do not interact (MUC4 and ZEB-1) were used to demonstrate the absence of artifactual PLA spots. Zooms are performed in the area delimited by a white square. PLA spots are shown in red and nuclei were stained with DAPI (blue). Scale bars = 20 µm. Arrows highlight example of PLA spots.

Figure S7. **Localization of *MUC4* mRNAs in Sh1 cells.** PolyA mRNA or *MUC4* specific transcripts were detected by FISH using either a Cy3.5 oligodT 40 mer or a Cy3.5 *MUC4* specific 48 mer probe (red). Gal-3 was detected by immunofluorescence (green). Nuclei were stained by DAPI (blue). Co-localization was assessed by merging the images (Merge). As expected, Sh1 expressed low levels of Gal-3 and *MUC4* transcripts. Scale bars: 10 µm.

Figure S8. Specificity control: *MUC4* mRNA and Gal-3 immunostainings performed on Sc cells previously treated by RNAse A (0.1 mg/ml, 30 min, 37°C). PolyA mRNA or *MUC4* specific transcripts were detected by FISH using either a Cy3.5 oligodT 40 mer or a Cy3.5 *MUC4* specific 48 mer probe (red). Gal-3 was detected by immunofluorescence (green). Nuclei were stained by DAPI (blue). Co-localization was assessed by merging the images (Merge). As expected, degradation of mRNAs by RNAse A treatment prevents the occurrence of a red fluorescence with the RNA probe, thus demonstrating the specificity of the signal observed in Figure 6 and S6. Scale bars: 10 µm.

Figure S9.PLA Xrn1+Gal-3

Grey scale image of the PLA interaction described in Figure 7.

Figure S10.PLA DDX6+Gal-3

Grey scale image of the PLA interaction described in Figure 7.

Figure S11.PLA G3P1+Gal-3

Grey scale image of the PLA interaction described in Figure 8b.

Figure S12.PLA eIF2α+Gal-3

Grey scale image of the PLA interaction described in Figure 8b.

Figure S1

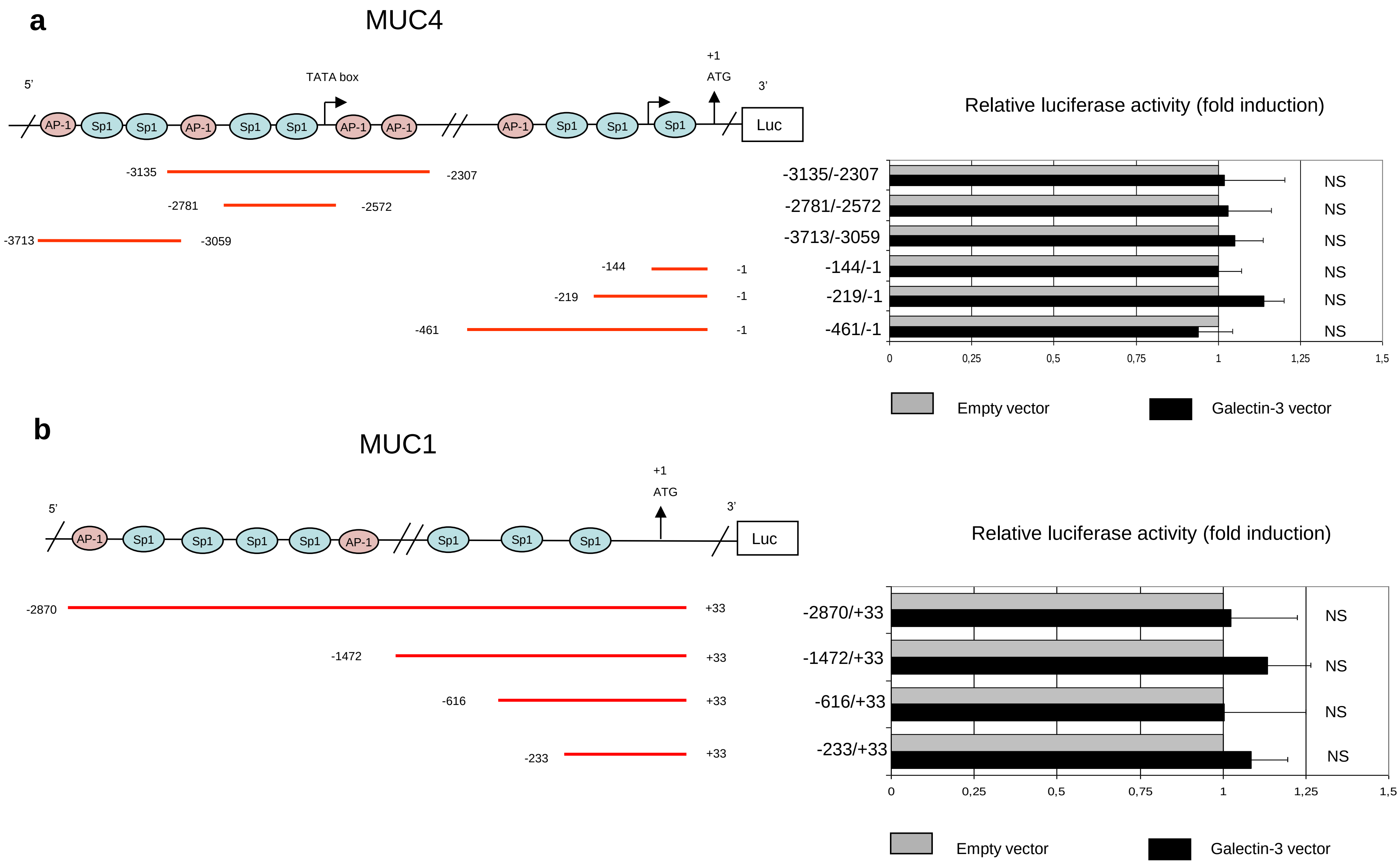

a
MUC4
5'
TATA box
+1
ATG
3'
AP-1
Sp1
Luc
-3135
-2307
-2781
-2572
-3713
-3059
-144
-1
-219
-1
-461
-1
Relative luciferase activity (fold induction)
-3135/-2307
-2781/-2572
-3713/-3059
-144/-1
-219/-1
-461/-1
NS
0
0,25
0,5
0,75
1
1,25
1,5
Empty vector
Galectin-3 vector
b
MUC1
5'
+1
ATG
3'
AP-1
Sp1
Luc
-2870
+33
-1472
+33
-616
+33
-233
+33
Relative luciferase activity (fold induction)
-2870/+33
-1472/+33
-616/+33
-233/+33
NS
0
0,25
0,5
0,75
1
1,25
1,5
Empty vector
Galectin-3 vector

Figure S2

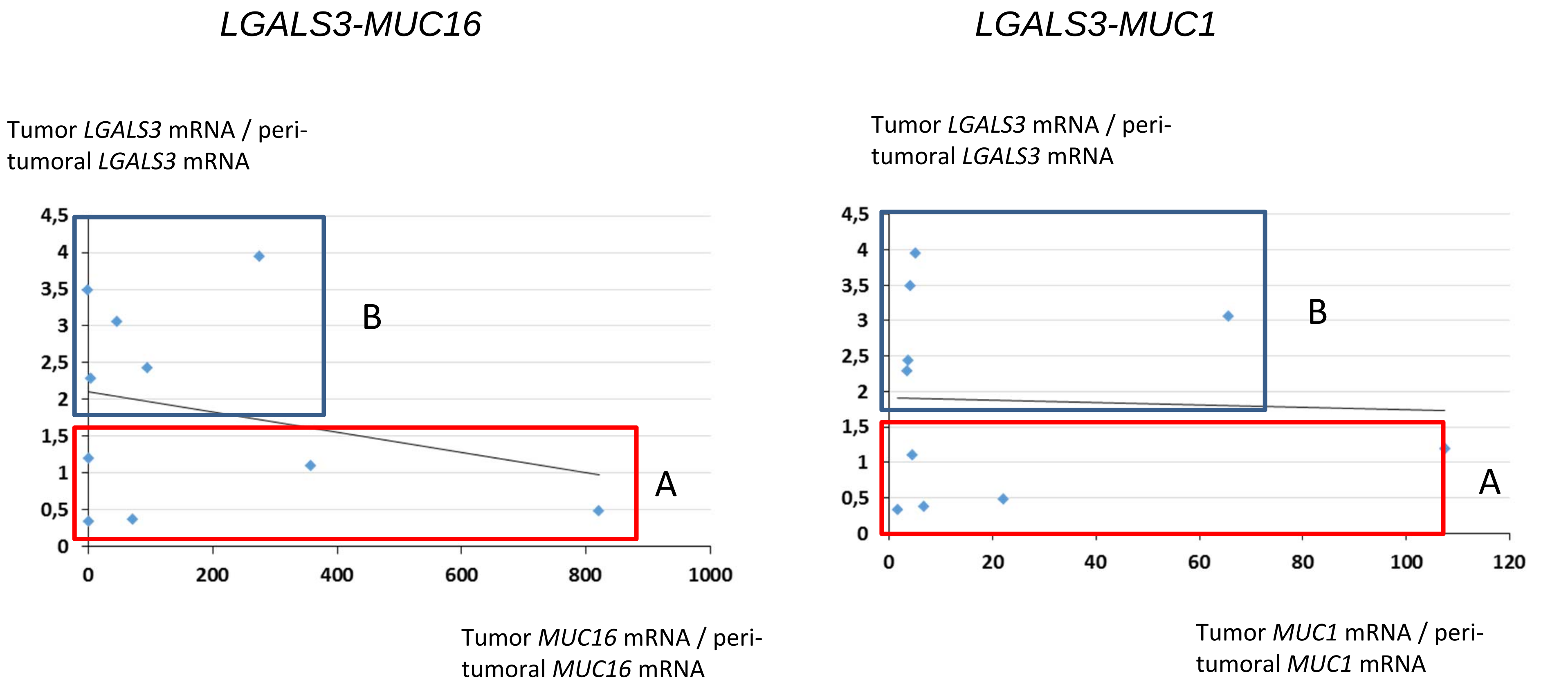

LGALS3-MUC16
Tumor LGALS3 mRNA / peri-tumoral LGALS3 mRNA
4,5
4
3,5
3
2,5
2
1,5
1
0,5
0
0
200
400
600
800
1000
B
A
Tumor MUC16 mRNA / peri-tumoral MUC16 mRNA
LGALS3-MUC1
Tumor LGALS3 mRNA / peri-tumoral LGALS3 mRNA
4,5
4
3,5
3
2,5
2
1,5
1
0,5
0
0
20
40
60
80
100
120
B
A
Tumor MUC1 mRNA / peri-tumoral MUC1 mRNA

Figure S3

Homo sapiens MUC4 gene, 3' flanking region (AJ010901.1)

```
      TAC TTC AGA TGC GAT GGC TAC AAG GGC TAC GAC CTG GTC TAC AGC CCC CAG
       Y   F   R   C   D   G   Y   K   G   Y   D   L   V   Y   S   P   Q
AGC GGC TTC ACC TGC GTG TCC CCG TGC AGT AGG GGC TAC TGT GAC CAT GGA GGC CAG TGC
 S   G   F   T   C   V   S   P   C   S   R   G   Y   C   D   H   G   G   Q   C
CAG CAC CTG CCC AGT GGG CCC CGC TGC AG-INTRON 24-C TGT GTG TCC TTC TCC ATC TAC
 Q   H   L   P   S   G   P   R   C   S            C   V   S   F   S   I   Y
ACG GCC TGG GGC GAG CAC TGT GAG CAC CTG AGC ATG AAA CTC GAC GCG TTC TTC GGC ATC
 T   A   W   G   E   H   C   E   H   L   S   M   K   L   D   A   F   F   G   I
TTC TTT GGG GCC CTG GGC GGC CTC TTG CTG CTG GGG GTC GGG ACG TTC GTG GTC CTG CGC
 F   F   G   A   L   G   G   L   L   L   L   G   V   G   T   F   V   V   L   R

TTC TGG GGT TGC TCC GGG GCC AGG TTC TCC TAT TTC CTG AAC TCA GCT GAG GCC TTG CCT
 F   W   G   C   S   G   A   R   F   S   Y   F   L   N   S   A   E   A   L   P

TGAAGGGGCAGCTGTGGCCTAGGCTACCTCAAGACTCACCTCATCCTTACCGCACATTTAAGGCGCCATTGCTTTTGG
 X                                                       ARE1           MIR 150

GAGACTGGAAAAGGGAAGGTGACTGAAGGCTGTCAGGATTCTT|CAAGGAGAATGAATACTGGGAATCAAGACAAGACT
                           CARE PROBE START
ATACCTTATCCATAGGCGCAGGTGCACAGGGGGAGGCCATAAAGATCAAACATGCATGGATGGGTCCTCACGCAGACAC
             MIR 210                  CA-REPEAT
ACCCACAGAAGGACACTAGCCTGTGCACGCGCGCGTGCACACACACACACACACACACGAGTTCATAATGTGGTGATGG
        AS2                                      AS1


CCCTAAGTTAAGCAAAATGCTTCTGCACACAAAACTCTCTGGTTTACTTCAAATTAACTCTATTTAAATAAAGTCTCTC
                                                   "ARE-LIKE"     ARE2
          3866
TGACTTTTTGTGTCTCCAAAAA
     ARE3   CARE PROBE STOP
```

Figure S4

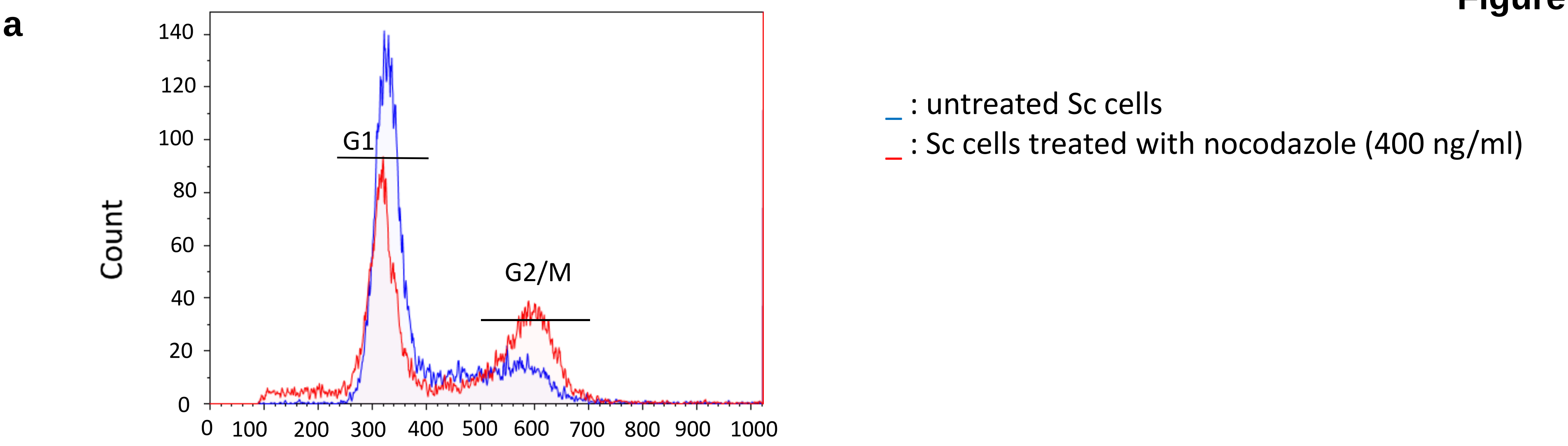

a
Count
140
120
100
80
60
40
20
0
0 100 200 300 400 500 600 700 800 900 1000
G1
G2/M
_ : untreated Sc cells
_ : Sc cells treated with nocodazole (400 ng/ml)

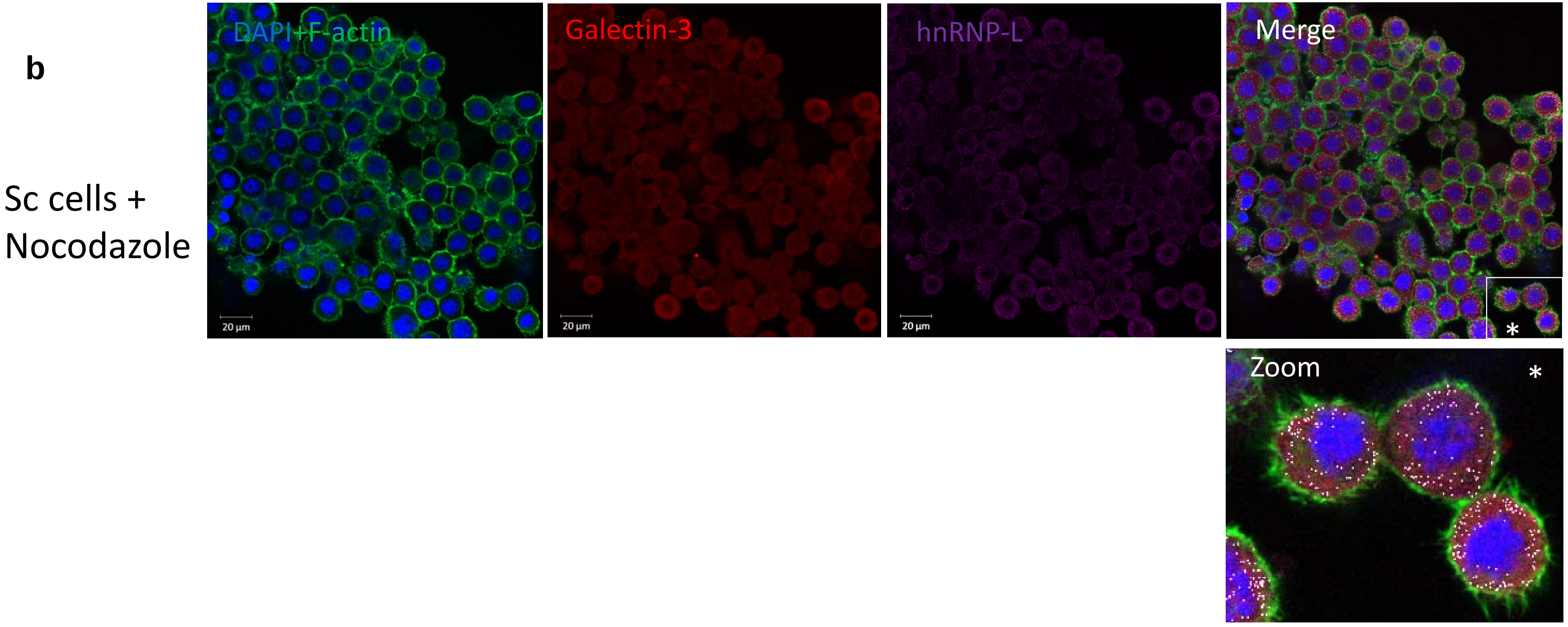

b
Sc cells + Nocodazole
DAPI+F-actin
Galectin-3
hnRNP-L
Merge
20 µm
20 µm
20 µm
*
Zoom
*

Figure S5

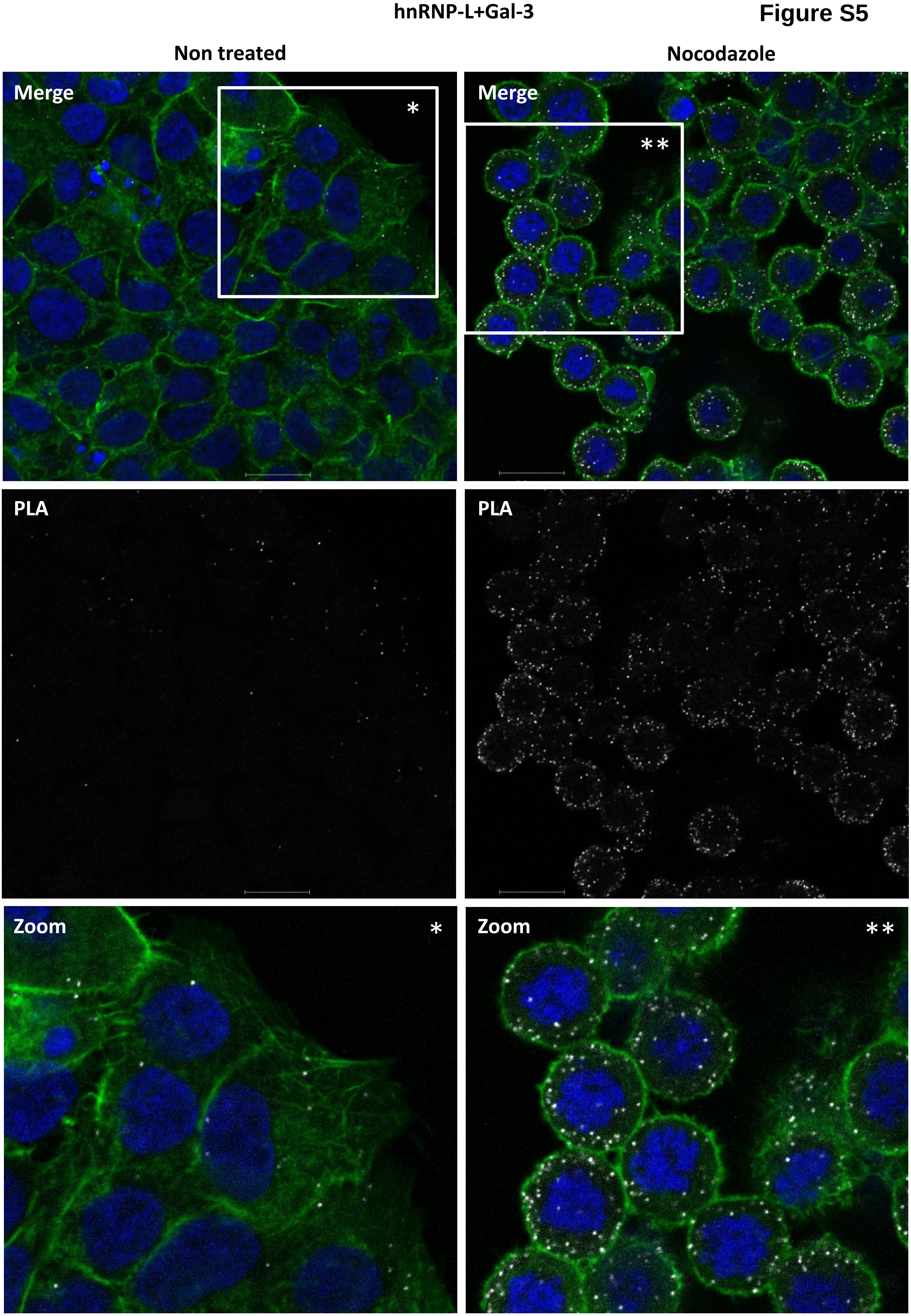

hnRNP-L+Gal-3
Non treated
Nocodazole
Merge
*
Merge
**
PLA
PLA
Zoom
*
Zoom
**

Figure S6

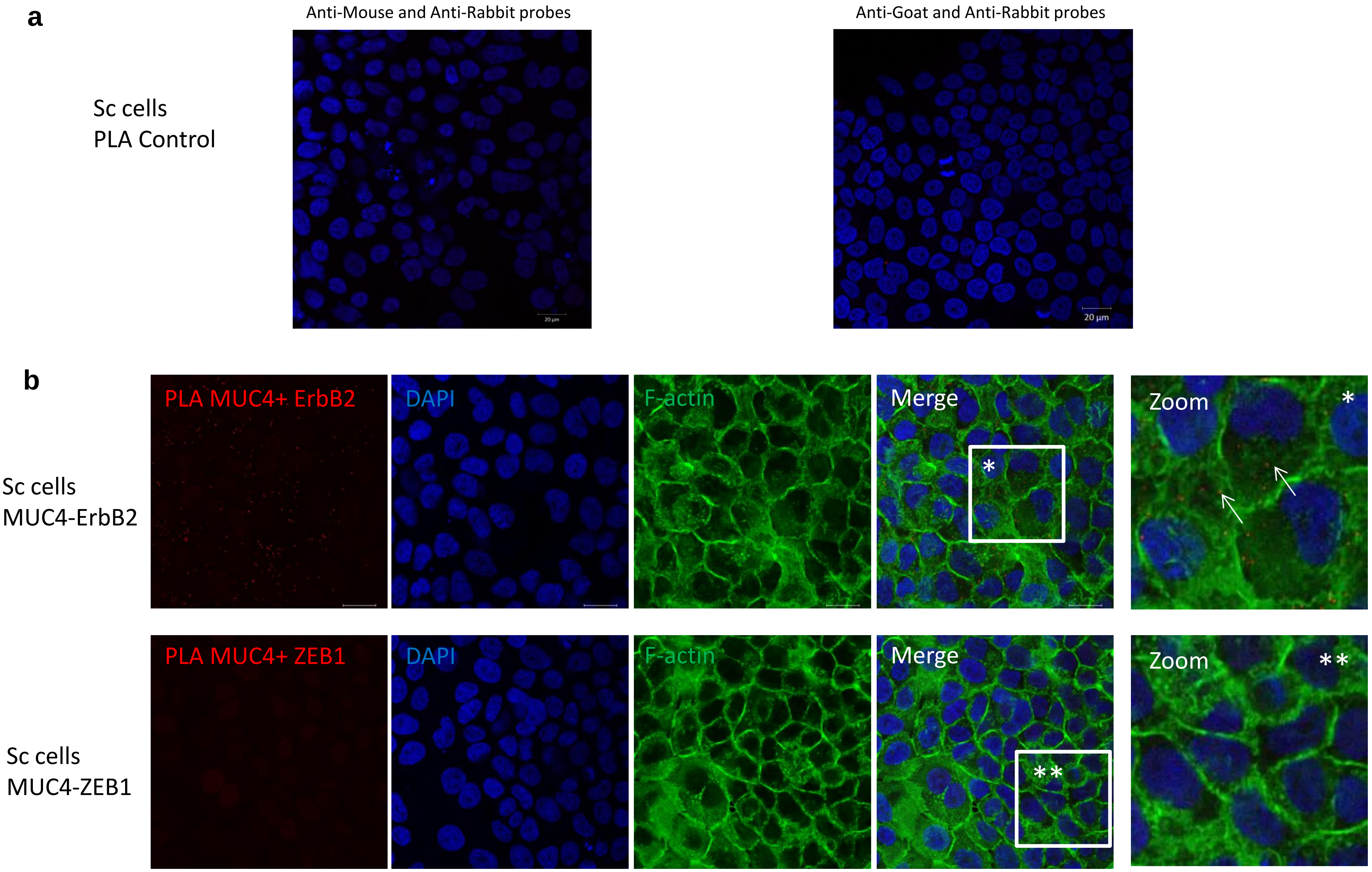
a
Anti-Mouse and Anti-Rabbit probes
Anti-Goat and Anti-Rabbit probes
Sc cells
PLA Control
b
PLA MUC4+ ErbB2
DAPI
F-actin
Merge
*
Zoom
*
Sc cells
MUC4-ErbB2
PLA MUC4+ ZEB1
DAPI
F-actin
Merge
**
Zoom
**
Sc cells
MUC4-ZEB1

Figure S7

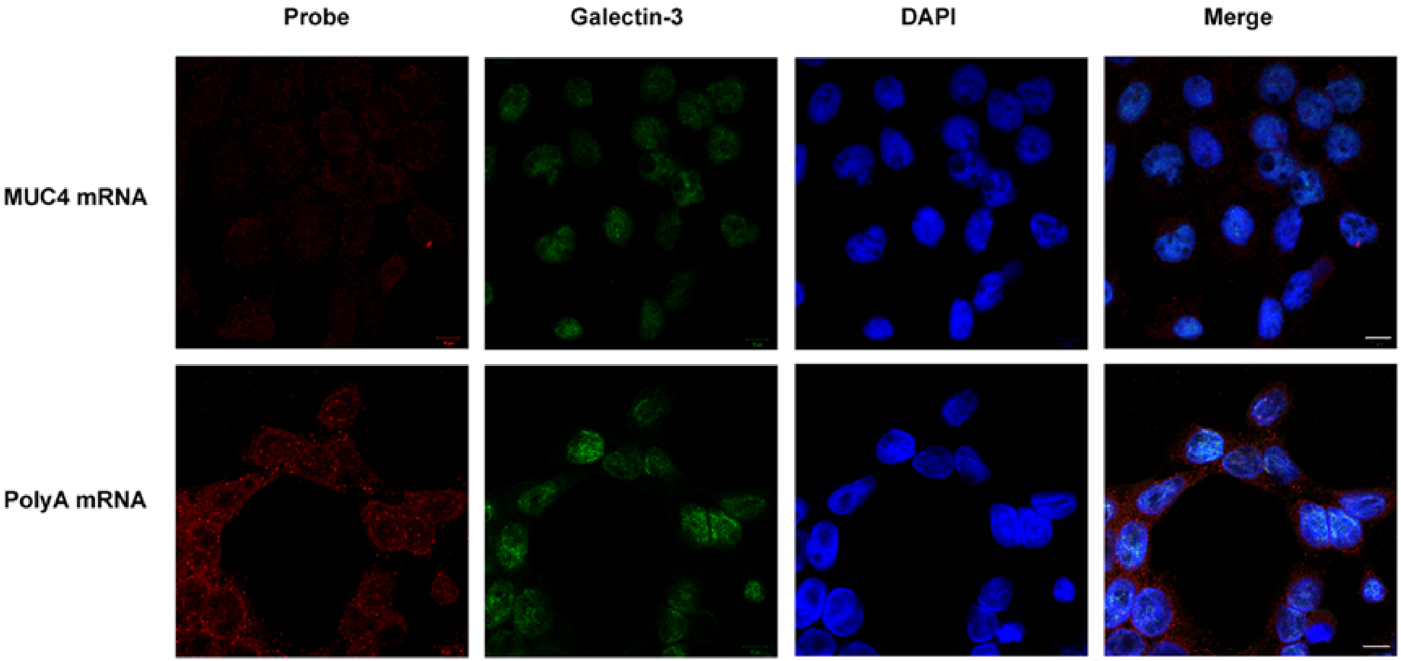

Figure S8

+RNAse treatment

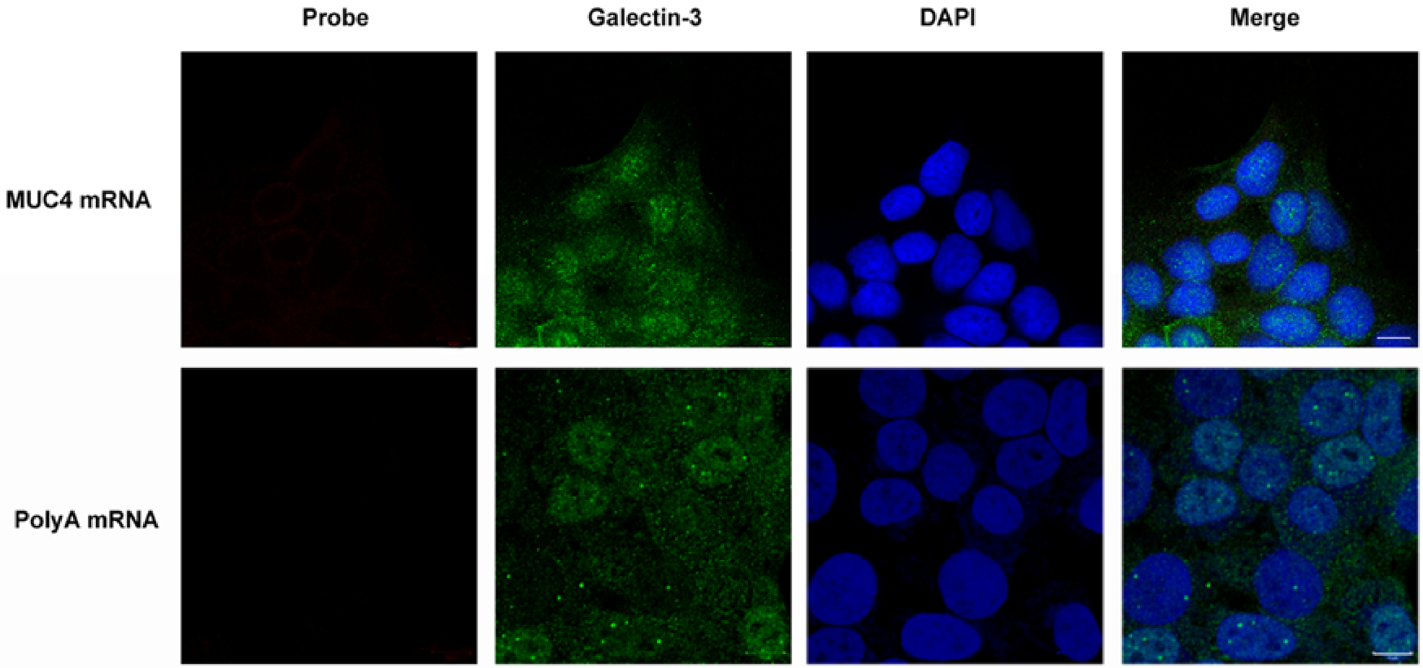

Xrn1+Gal-3

Figure S9

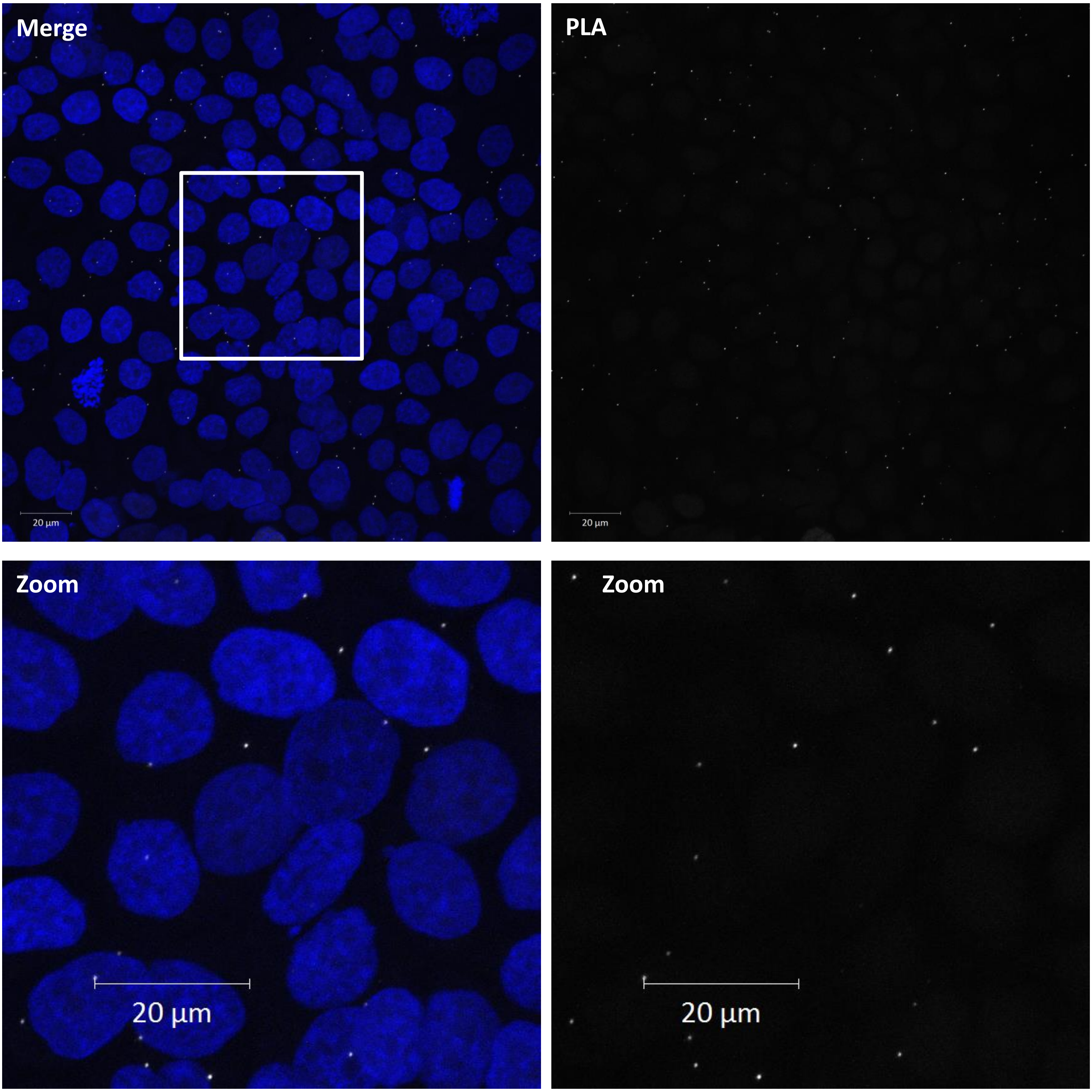

DDX6+Gal-3

Figure S10

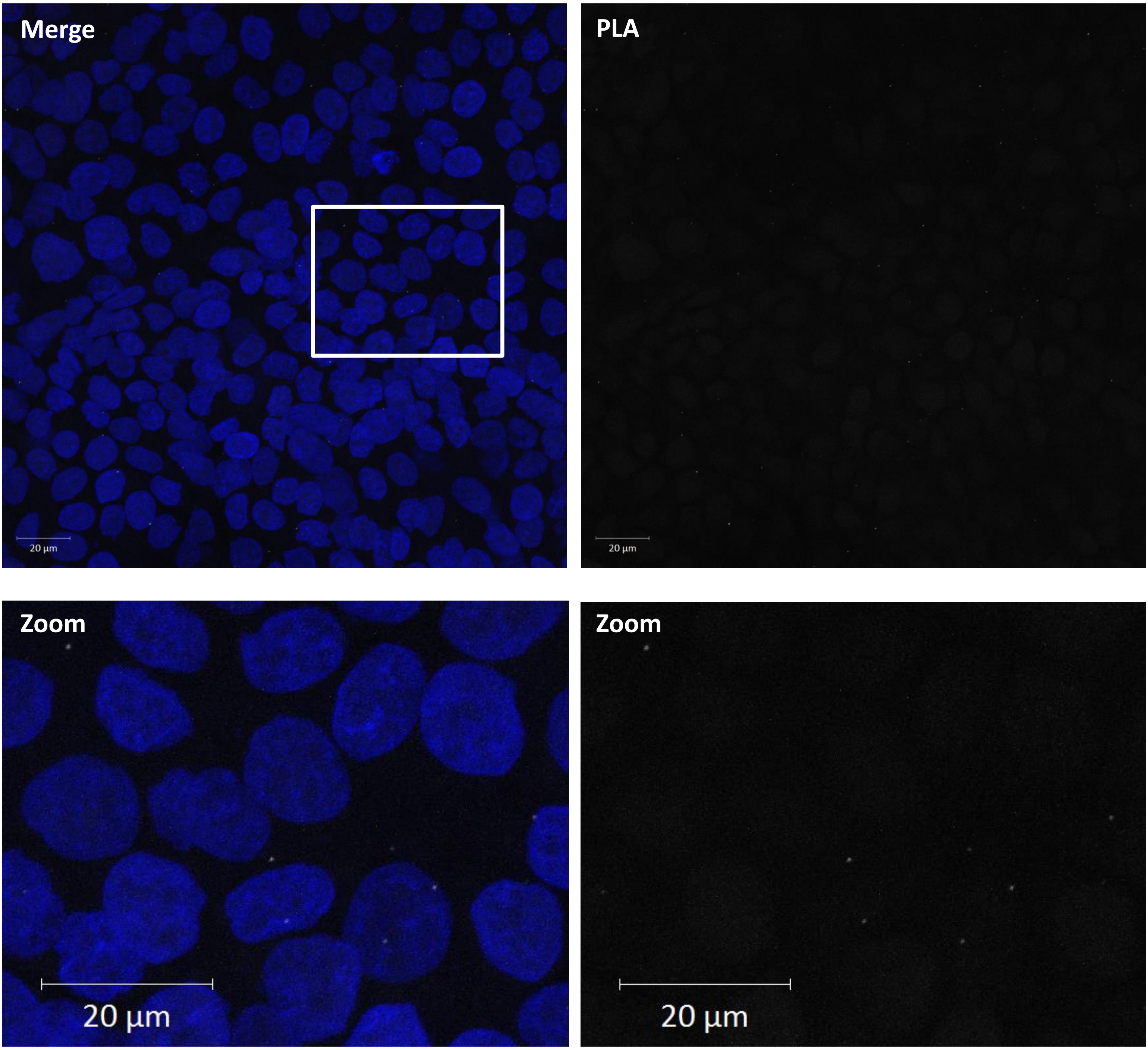

Figure S11

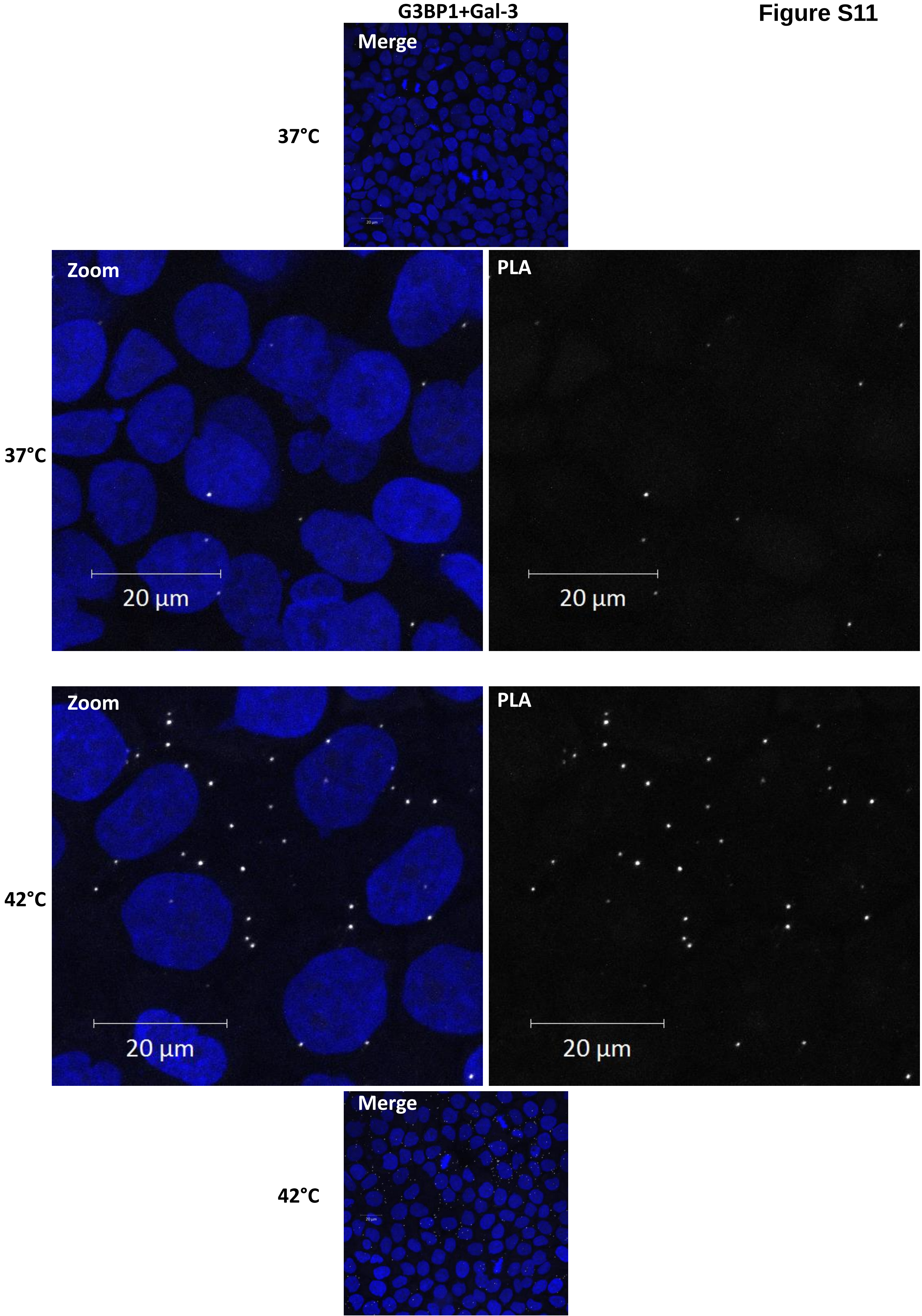
G3BP1+Gal-3
Merge
37°C
Zoom
PLA
20 µm
20 µm
Zoom
PLA
42°C
20 µm
20 µm
Merge
42°C

Figure S12

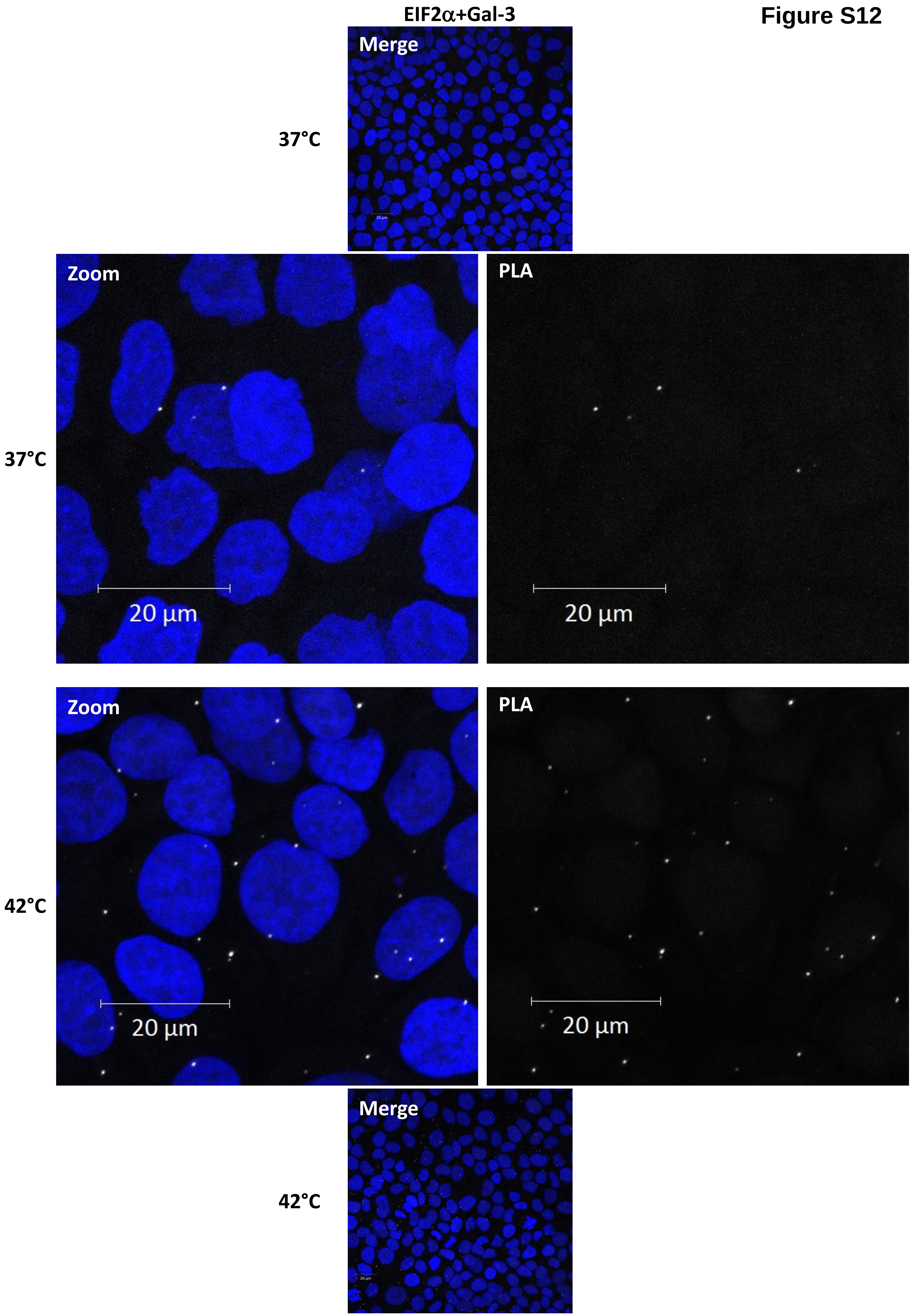
EIF2α+Gal-3
Merge
37°C
Zoom
PLA
37°C
20 µm
20 µm
Zoom
PLA
42°C
20 µm
20 µm
Merge
42°C
